# *Plasmodium knowlesi* Infection Is Associated With Elevated Circulating Biomarkers of Brain Injury and Endothelial Activation

**DOI:** 10.1093/infdis/jiae553

**Published:** 2024-12-10

**Authors:** Cesc Bertran-Cobo, Elin Dumont, Naqib Rafieqin Noordin, Meng-Yee Lai, William Stone, Kevin K A Tetteh, Chris Drakeley, Sanjeev Krishna, Yee-Ling Lau, Samuel C Wassmer

**Affiliations:** Department of Infection Biology, London School of Hygiene and Tropical Medicine, United Kingdom; Department of Psychiatry and Mental Health, University of Cape Town, South Africa; Neuroscience Institute, University of Cape Town, South Africa; Department of Infection Biology, London School of Hygiene and Tropical Medicine, United Kingdom; Department of Parasitology, Faculty of Medicine, Universiti Malaya, Kuala Lumpur, Malaysia; Department of Parasitology, Faculty of Medicine, Universiti Malaya, Kuala Lumpur, Malaysia; Department of Infection Biology, London School of Hygiene and Tropical Medicine, United Kingdom; Department of Infection Biology, London School of Hygiene and Tropical Medicine, United Kingdom; Department of Infection Biology, London School of Hygiene and Tropical Medicine, United Kingdom; Department of Parasitology, Faculty of Medicine, Universiti Malaya, Kuala Lumpur, Malaysia; Institut Für Tropenmedizin, Eberhard Karls Universität Tübingen, Germany; Centre de Recherches Médicales de Lambaréné, Gabon; Clinical Academic Group in Institute for Infection and Immunity, St George's University of London, United Kingdom; Department of Parasitology, Faculty of Medicine, Universiti Malaya, Kuala Lumpur, Malaysia; Department of Infection Biology, London School of Hygiene and Tropical Medicine, United Kingdom

**Keywords:** *Plasmodium knowlesi*, malaria, brain, vascular dysfunction, Malaysia

## Abstract

**Background:**

Malaria remains a major public health concern with substantial morbidity and mortality worldwide. In Malaysia, the emergence of *Plasmodium knowlesi* has led to a surge in zoonotic malaria cases and deaths in recent years. Signs of cerebral involvement have been observed in a noncomatose, fatal case of knowlesi infection, but the potential impact of this malaria species on the brain remains unexplored. To address this gap, we investigated circulating levels of brain injury, inflammation, and vascular biomarkers in a cohort of knowlesi-infected patients and controls.

**Methods:**

Archived plasma samples from 19 Malaysian patients with symptomatic knowlesi infection and 19 healthy, age-matched controls were analyzed. Fifty-two biomarkers of brain injury, inflammation, and vascular activation were measured. Wilcoxon tests were used to examine group differences, and biomarker profiles were explored through hierarchical clustering heatmap analysis.

**Results:**

Bonferroni-corrected analyses revealed significantly elevated brain injury biomarker levels in knowlesi-infected patients, including S100B (*P* < .0001), Tau (*P* = .0007), UCH-L1 (*P* < .0001), αSyn (*P* < .0001), Park7 (*P* = .0006), NRGN (*P* = .0022), and TDP-43 (*P* = .005). Compared to controls, levels were lower in the infected group for BDNF (*P* < .0001), CaBD (*P* < .0001), CNTN1 (*P* < .0001), NCAM-1 (*P* < .0001), GFAP (*P* = .0013), and KLK6 (*P* = .0126). Hierarchical clustering revealed distinct group profiles for brain injury and vascular activation biomarkers.

**Conclusions:**

Our findings highlight for the first time a potential impact of *P knowlesi* infection on the brain, with specific changes in cerebral injury and endothelial activation biomarker profiles. Further studies are warranted to investigate the pathophysiology and clinical significance of these altered markers, through neuroimaging and long-term neurocognitive assessments.

## Teaser

*Plasmodium knowlesi* infection results in plasma biomarker changes that are associated with neurovascular injury. These findings provide insight into the pathophysiology of knowlesi malaria in humans and suggests potential long-term effects on brain health that warrant further investigations.

Malaria is a life-threatening, vector-borne infection caused by parasites of the genus *Plasmodium*, that led to an estimated 249 million cases worldwide and approximately 608 000 deaths in 2022 [1]. Malaria is endemic in Southeast Asia, where it poses a significant public health challenge. In Malaysia, *Plasmodium falciparum* and *Plasmodium vivax* have historically been the species responsible for most malaria infections and deaths, but thanks to national efforts in eradicating the disease, no indigenous cases have been reported since 2018 [1]. However, an emerging concern in the country is *Plasmodium knowlesi* (*Pk*), a zoonotic species that has become a significant contributor to malaria infections in humans locally [2, 3], with a total of 19 625 *Pk* cases and 57 deaths reported in Malaysia since 2017, including 2500 cases and 9 deaths in 2022 alone [1]. Malaysia accounts for most *Pk* infections globally, and this species is currently the major cause of human malaria in the country [4, 5].

One of the most severe manifestations of *P falciparum* infection is cerebral malaria, a life-threatening neurological complication characterized by coma [6] and pathological hallmarks such as petechial hemorrhages and parasite sequestration in the brain vasculature [7]. Neurocognitive sequelae are frequent in survivors and can persist long after the infection has been treated [8, 9]. While cerebral malaria is predominantly associated with *P falciparum* [6], recent case reports have highlighted instances of cerebral involvement and severe neurological complications in patients infected with other *Plasmodium* species [10–13]. A high proportion of severe *Pk* infections has been reported in Southeast Asia [5, 14], as well as fatal cases [13–15]. A recent study also showed that, similarly to *P falciparum*, *Pk*-infected erythrocytes are able to bind to endothelial cells [16]. Postmortem findings in one fatal case of severe *Pk* malaria revealed brain pathology features similar to those seen in fatal falciparum cerebral malaria, including *Pk*-infected erythrocyte sequestration in the microvasculature [13], suggesting that *Pk* malaria may also affect the brain. Remarkably, coma was not observed in this patient [13], which contrasts with the World Health Organization definition of cerebral malaria caused by *P falciparum* [6].

We recently reported that patients with severe and uncomplicated falciparum malaria—and therefore without coma—have a wide range of brain changes detected using magnetic resonance imaging (MRI) [17, 18]. Severe malaria patients from our cohort had elevated plasma levels of the neurotrophic factor S100 calcium-binding protein β (S100B), a biomarker associated with central nervous system insults [19], which correlated with brain MRI features typically found in cerebral malaria [17]. Overall, our findings suggest that there is a frequent and unrecognized impact of malaria infection on the brain in falciparum malaria, irrespective of coma. These effects are likely aggravated by acute kidney injury (AKI), with potential long-lasting repercussions on quality of life and productivity in survivors [20].

Despite the similar pathologies reported between cerebral malaria and fatal *Pk* infection [13] and the high incidence of kidney dysfunction in severe *Pk* malaria [21], the potential impact of *Pk* infection on the human brain remains largely unexplored. To bridge this important knowledge gap, we quantified plasma biomarkers of brain injury, vascular activation, and inflammation in a cohort of Malaysian *Pk*-infected patients and healthy controls.

## MATERIALS AND METHODS

### Participants and Samples

**Figure 1.**
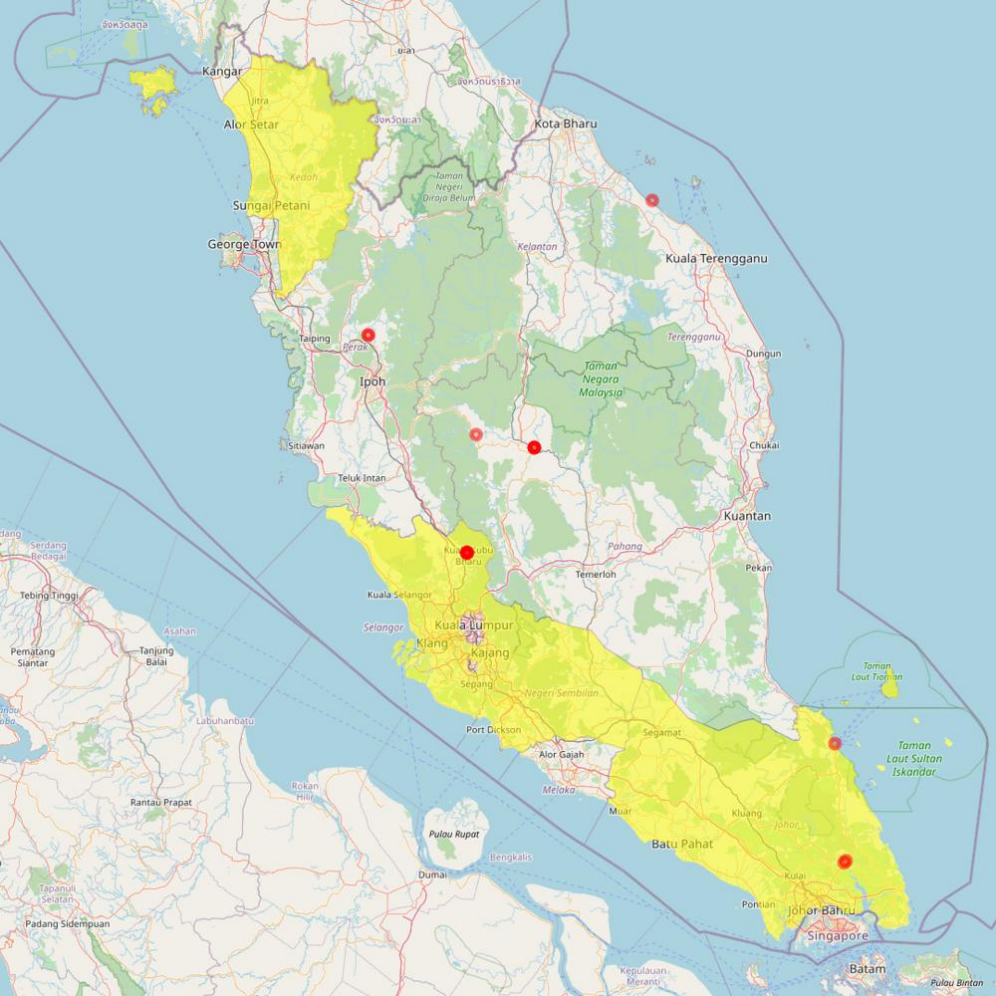
Sample collection sites. Samples from *Plasmodium knowlesi*–infected patients were collected from government hospitals and private clinics in Johor, Selangor, Pahang, Perak, and Trengganu states (dots). Samples from community-matched, age-matched uninfected controls were collected via active sample screening of communities in Johor, Selangor, Negeri Sembilan, and Kedah (highlighted areas). Map was created in R with RStudio using Leaflet (https://leafletjs.com/) and OpenStreetMap (https://www.openstreetmap.org). An interactive version of the map can be found in GitHub (https://github.com/Cescualito/LSHTM_Wassmer_Pknowlesi_Malaysia).

This study leveraged archived plasma samples from patients aged ≥18 years with malaria symptoms attending either a government hospital or private clinic in Johor, Selangor, Pahang, Perak, and Trengganu states between December 2019 and January 2023 (Figure 1). Recruitment of community-matched, age-matched, uninfected controls was conducted via active screening of individuals from communities in Johor, Selangor, Negeri Sembilan, and Kedah. Subjects were approached for participation if they had no fever, were aged ≥18 years, and were part of any of the considered high-risk groups (defined as individuals working in proximity with forest and forest fringes) [22]. Data collection procedures are described elsewhere [23]. For this study, a total of 38 serum samples from 19 infected patients and 19 healthy controls were analyzed (Supplementary Methods).

### Biomarker Panel Selection

Our biomarker panel was designed to include 3 subpanels (Supplementary Methods). First, a brain injury subpanel included 9 biomarkers directly selected for their established roles in malaria-related brain injury across *Plasmodium* species (Supplementary Table 1). Additional 12 brain injury biomarkers were included due to their relevance in neurodegeneration and brain injury, with a focus on neuroinflammatory processes that may overlap with those reported in malaria [8, 9]. Second, an infection and immune activation subpanel comprised 21 biomarkers, selected based on their documented associations with immune responses in malaria infection (Supplementary Table 2). Last, an endothelial activation subpanel consisted of 10 vascular biomarkers, chosen through a similar evidence-based approach (Supplementary Table 3).

### Measurement of Plasma Biomarker Levels via Luminex and Simoa Assays

Thirty-eight biomarkers were analyzed using the customizable Human Luminex Discovery Assay (LXSAHM, R&D Bio-Techne): alpha-synuclein (αSyn), amyloid-β precursor protein (APP), angiopoietin-1 (Ang-1), angiopoietin-2 (Ang-2), bone morphogenetic protein 9 (BMP-9), calbindin D (CaBD), chemokine ligand 2 (CCL2), chemokine ligand 4 (CCL4), chemokine ligand 5 (CCL5), chemokine ligand 18 (CCL18), contactin-1 (CNTN1), C-reactive protein (CRP), enolase 2/neuron-specific enolase (ENO2/NSE), fetuin A, granulocyte-macrophage colony-stimulating factor (GM-CSF), interferon gamma (IFN-γ), intercellular adhesion molecule 1 (ICAM-1), interleukin 1 alpha (IL-1α), interleukin 1 beta (IL-1β), interleukin 1RA (IL-1RA), interleukin 2 (IL-2), interleukin 4 (IL-4), interleukin 6 (IL-6), interleukin 8 (IL-8), interleukin 10 (IL-10), interleukin 17 (IL-17A), lipocalin-2 (neutrophil gelatinase–associated lipocalin [NGAL]), myeloperoxidase (MPO), osteopontin (OPN), parkinsonism-associated deglycase (Park7), platelet-derived growth factor AA (PDGF-AA), platelet-derived growth factor BB (PDGF-BB), receptor for advanced glycation end products (RAGE), serine proteinase inhibitor clade E1 (Serpin E1), tumor necrosis factor alpha (TNF-α), vascular cell adhesion molecule (VCAM-1), vascular endothelial growth factor (VEGF), and von Willebrand factor A2 domain (vWF-A2).

Additionally, 10 biomarkers were analyzed using the Neuroscience 18-plex Human ProcartaPlex Panel assay (Thermo Fisher Scientific/Invitrogen, EPX180-15837-901): amyloid-β (1-42) (Aβ_(1-42)_), brain-derived neurotrophic factor (BDNF), kallikrein 6 (KLK6), migration inhibitory factor (MIF), nerve growth factor beta (NGF-β), neural cell adhesion molecule (NCAM-1), neurogranin (NRGN), S100B, TAR DNA-binding protein 43 (TDP-43), and chitinase-3-like protein 1 (YKL-40).

For these assays, serum levels of biomarkers were measured using a MAGPIX bioanalyzer (Diasorin), according to the manufacturer's instructions. Singlicate measurements were taken of each sample. Sample concentrations were extrapolated from a standard curve, fitted using a 6-parameter logistic algorithm.

Last, 4 brain injury biomarkers were analyzed using a highly sensitive single molecule immunoassay using Human Neurology 4-Plex A assay (Quanterix, 102153). Neurofilament light (NfL), total Tau protein (Tau), glial fibrillary acidic protein (GFAP), and ubiquitin carboxyl-terminal hydrolase L1 (UCH-L1) concentrations were measured on a Simoa HD-X Analyzer (Quanterix), according to the manufacturer's instructions. In brief, samples were thawed at 21°C and centrifuged at 10 000 relative centrifugal force for 5 minutes at the same temperature. Onboard the instrument, samples were diluted 1:4 with sample diluent and bound to paramagnetic beads coated with specific capture antibodies. Beads bound to these markers were then incubated with biotinylated detection antibodies in turn conjugated to streptavidin-β-galactosidase complex that acts as a fluorescent tag. Subsequent hydrolysis reaction with a resorufin β-D-galactopyranoside substrate produced a fluorescent signal proportional to the concentration of GFAP, NfL, Tau, and UCH-L1. Analyte concentrations were extrapolated from a standard curve, fitted using a 4-parameter logistic algorithm.

### Serological Assessment of Malaria Exposure via Luminex Immunoassay

As past infections with *Plasmodium* spp could also influence observed biomarker levels across participants, total immunoglobulin G (IgG) antibody responses of all *Pk*-infected patients and healthy controls were measured using a multiplex bead-based immunoassay developed for Luminex xMAP technology to assess previous exposure to *Pk* as well as to *P falciparum*, *P vivax*, *Plasmodium malariae*, and *Plasmodium ovale* (Supplementary Methods, Supplementary Table 4).

### Statistical Analysis

Sociodemographic characteristics of the participants were reported as mean ± standard deviation for continuous data, or absolute frequencies (%) for categorical data. Continuous data were assessed for normality using Shapiro-Wilk tests. Comparisons between *Pk*-infected cases and healthy controls were made using *t* tests or Wilcoxon tests for normally and non–normally distributed continuous data, respectively, and χ^2^ tests for categorical data.

Differences in levels of individual biomarkers between *Pk*-infected patients and controls were examined using 2-tailed *t* tests and Wilcoxon tests for normally and non–normally distributed data, respectively. Bonferroni correction for multiple comparisons was applied. Correlation matrixes were used to visualize the relationships between biomarker levels in both groups, with previous data scaling. Any potential correlations between biomarker levels and demographic and clinical parameters were evaluated using Spearman rank correlation coefficient.

To elucidate potential profiles of biomarker levels that could distinguish between infected patients and controls, a hierarchical clustering heatmap analysis was performed, prior to scaling of biomarker data. This provided a visual representation of the relationships between individuals based on the similarity of their biomarker profiles. The clustering analysis was applied separately to each biomarker subpanel.

To assess malaria exposure, a hierarchical clustering heatmap was similarly performed to allow visual comparison of individual antibody responses to *Pk-*specific antigen profiles across study participants. Comparisons of group means of mean fluorescence intensity responses between *Pk*-infected cases and healthy controls were conducted using the nonparametric Kruskal-Wallis test with Dunn correction for multiple comparisons.

Statistical analyses were performed in R (version 4.3.1) with RStudio software (version 2023.09.1-494), and data were visualized in R (version 4.3.1) with RStudio software (version 2023.09.1-494) and GraphPad Prism 10 software. *P* values of <.05 (2-tailed) were considered statistically significant.

## RESULTS

### Cohort Characteristics

All participants were male (N = 38 [100%]). Both *Pk*-infected patients and community-matched, age-matched uninfected controls had an average age of 39 (±15) years. In the *Pk*-infected group, patients presented with median parasitemia 15 200 parasites/μL (interquartile range 372,750–3,047,350).

### Concentrations of Individual Markers Between *Pk*-Infected Patients and Controls

#### Brain Injury Subpanel

**Figure 2.**
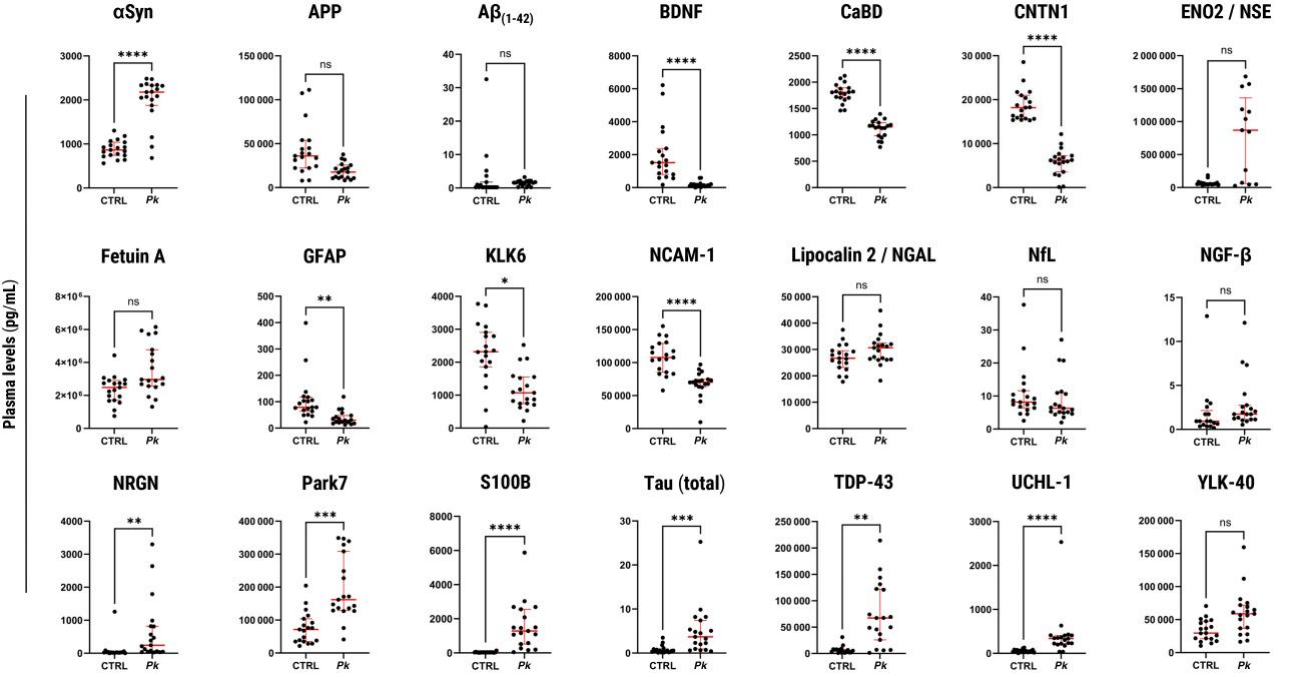
Group comparisons: levels of blood circulating biomarkers of brain injury. Wilcoxon test results: *P < .05; **P < .01; ***P < .001; ****P < .0001; ns, not significant. Abbreviations: αSyn, alpha-synuclein; Aβ_(1-42)_, amyloid-β (1-42); AHSG, alpha 2-HS glycoprotein; APP, amyloid-β precursor protein; BDNF, brain-derived neurotrophic factor; CaBD, calbindin D; CNS, central nervous system; CNTF, ciliary neurotrophic factor; CNTN1, contactin-1; CSF, cerebrospinal fluid; ENO2/NSE, enolase 2/neuron-specific enolase; FGF-21, fibroblast growth factor 21; GDNF, glial cell line–derived neurotrophic factor; GFAP, glial fibrillary acidic protein; KLK6, kallikrein 6/neurosin; NCAM-1, neural cell adhesion molecule; NGAL, neutrophil gelatinase–associated lipocalin (also known as lipocalin-2); NfL, neurofilament light chain; NGF-β, nerve growth factor beta; NRGN, neurogranin; Park7, parkinsonism-associated deglycase; S100B, S100 calcium-binding protein β; Tau, total Tau protein; Tau pT181, phosphorylated Tau protein; TDP-43, TAR DNA-binding protein 43; UCH-L1, ubiquitin carboxy-terminal hydrolase L1; YKL40, chitinase-3-like protein 1.

Bonferroni-corrected analyses revealed significantly higher plasma levels of the following brain injury biomarkers in the *Pk*-infected group, compared with uninfected controls: S100B (*P* < .0001), Tau (*P* = .0007), UCH-L1 (*P* < .0001), αSyn (*P* < .0001), Park7 (*P* = .0006), NRGN (*P* = .0022), and TDP-43 (*P* = .005). In contrast, levels of the following biomarkers were found to be significantly lower in the *Pk*-infected group when compared with uninfected controls: CaBD (*P* < .0001), CNTN1 (*P* < .0001), NCAM-1 (*P* < .0001), BDNF (*P* < .0001), GFAP (*P* = .0013), and KLK6 (*P* = .0126). Results obtained on CaBD and CNTN1 levels revealed clear cutoff values that allowed for subject classification based on infection status: All *Pk*-infected patients (19/19 [100%]) presented with plasma CaBD levels <1400 pg/mL or CNTN1 levels <12 500 pg/mL, whereas concentrations in all uninfected controls (19/19 [100%]) were >1400 pg/mL for CNTN1 and >15 000 pg/mL for CNTN1, respectively (Figure 2, Supplementary Results, Supplementary Table 5).

#### Infection and Immune Activation Subpanel

Group differences in plasma levels were found after correction for multiple comparisons. IL-1RA (*P* < .0001), IL-10 (*P* < .0001), and MPO levels (*P* = .0314) were significantly higher in the *Pk*-infected group when compared with their uninfected peers, whereas CCL4 (*P* < .0001), CCL5 (*P* < .0001), CRP (*P* = .0280), and RAGE levels (*P* = .0025) were significantly lower (Supplementary Figure 1, Supplementary Table 5).

#### Endothelial Activation Subpanel

Ang-2/Ang-1 ratios (*P* < .0001) and VCAM-1 levels (*P* = .0001) were significantly higher in *Pk*-infected patients compared with uninfected controls, whereas Ang-1 (*P* < .0001), BMP-9 (*P* < .0001), PDGF-AA and -BB (*P* < .0001), and Serpin E1 levels (*P* < .0001) were significantly lower (Supplementary Figure 2, Supplementary Table 5).

### Hierarchical Clustering of Samples Based on Biomarker Blood Levels

**Figure 3.**
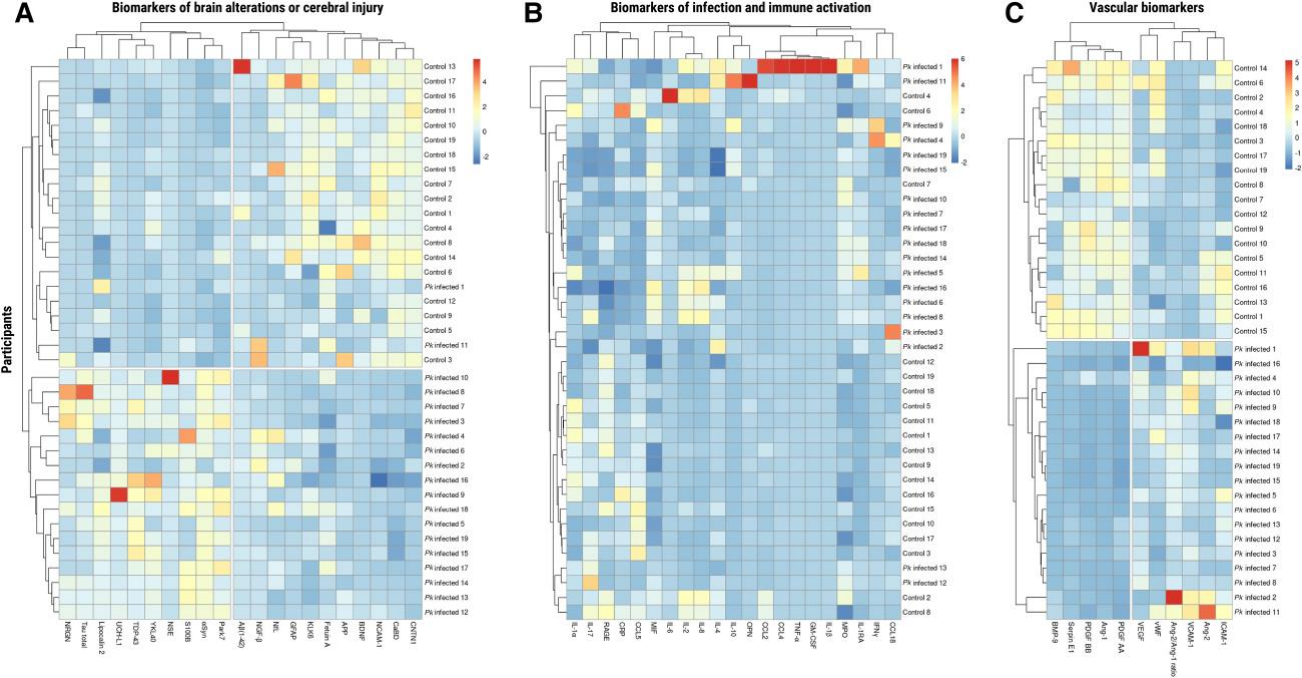
Hierarchical clustering heatmaps: distinct circulating biomarker profiles between groups. *A*, Biomarkers of brain alterations or cerebral injury. *B*, Biomarkers of infection and immune activation. *C*, Vascular biomarkers. Data are scaled. Abbreviations: αSyn, alpha-synuclein; Aβ_(1-42)_, amyloid-β (1-42); Ang-1, angiopoietin-1; Ang-2, angiopoietin-2; Ang-2/Ang-1, ratio between Ang-2 and Ang-1; APP, amyloid-β precursor protein; BDNF, brain-derived neurotrophic factor; BMP-9, bone morphogenetic protein 9; CaBD, calbindin D; CCL, chemokine (C-C motif) ligand; CNTN1, contactin-1; CRP, C-reactive protein; CSF, cerebrospinal fluid; ENO2/NSE, enolase 2/neuron-specific enolase; GFAP, glial fibrillary acidic protein; GM-CSF, granulocyte-macrophage colony-stimulating factor; ICAM-1, intercellular adhesion molecule 1; IFN-γ, interferon gamma; IL, interleukin; KLK6, kallikrein 6/neurosin; Lipocalin-2, neutrophil gelatinase–associated lipocalin; MIF, migration inhibitory factor; MPO, myeloperoxidase; NCAM-1, neural cell adhesion molecule; NfL, neurofilament light chain; NGF-β, nerve growth factor beta; NRGN, neurogranin; OPN, osteopontin; Park7, parkinsonism-associated deglycase; PDGF, platelet-derived growth factor; *Pk*, *Plasmodium knowlesi*; RAGE, receptor for advanced glycation end products; S100B, S100 calcium-binding protein β; Serpin E1, serine proteinase inhibitor clade E1; Tau, total Tau protein; TDP-43, TAR DNA-binding protein 43; TNF-α, tumor necrosis factor alpha; UCH-L1, ubiquitin carboxy-terminal hydrolase L1; VCAM, vascular cell adhesion molecule; VEGF, vascular endothelial growth factor; vWF-A2, von Willebrand factor (A2 domain); YKL40, chitinase-3-like protein 1.

Hierarchical clustering heatmap analyses revealed distinct group profiles for brain injury biomarkers (Figure 3*A*). Most infected individuals clustered together (17/19 [89.47%]), indicating a cohesive pattern of elevated levels of Park7, S100B, αSyn, and TDP-43. Two infected individuals exhibited atypical clustering with the healthy controls, suggesting a subgroup with a distinct biomarker profile. In the control group (19/19 [100%]), certain biomarkers, including BDNF, CaBD, CNTN1, and GFAP, displayed higher levels and clustered together, representing a baseline biomarker profile in healthy individuals. As mentioned, 2 infected individuals (2/19 [10.53%]) clustered with the control group in this category, indicating potential overlap or similarity in the levels of these specific biomarkers between infected and control individuals.

Clustering of vascular biomarkers revealed clear and distinctive group profiles (Figure 3*C*). In the control group (19/19 [100%]), a cohesive cluster formed by high levels of PDGF-AA, Ang-1, PDGF-BB, Serpin E1, and BMP-9 was observed. Conversely, in the infected group (19/19 [100%]), a distinct cluster of higher levels of VCAM-1 and the Ang-2/Ang-1 ratio was identified. Last, clustering of biomarkers associated with infection and immune activation did not reveal distinct separation between the 2 groups (Figure 3*B*, Supplementary Figure 3).

## DISCUSSION

Our study sheds light on the potential neurological implications of *Pk* infection in Malaysia. Our investigation of circulating levels of brain injury, inflammation, and vascular biomarkers in *Pk*-infected patients compared to healthy controls uncovered alterations potentially associated with cerebral injury.

Circulating levels of S100B, Tau, UCH-L1, BDNF, and NCAM-1 have been documented in previous malaria studies. S100B, an abundant neurotrophic factor predominantly expressed in astrocytes, is a biomarker of blood-brain barrier (BBB) barrier permeability and central nervous system injury [24]. Our group reported increased plasma levels of S100B in Indian patients with falciparum severe malaria compared to uncomplicated cases [17] (Table 1). Similarly, levels of microtubule-associated protein Tau, a neuropathological hallmark of Alzheimer disease and a biomarker for brain injury [25], were found to be elevated in the plasma of Ugandan children with cerebral malaria compared to uninfected controls and correlated with mortality and neurocognitive impairment [26]. Plasma levels of the ubiquitin-protein hydrolase UCH-L1, a marker of neuronal damage [27], were significantly higher in Ugandan children with cerebral malaria compared with asymptomatic community children and were linked to BBB dysfunction and cognitive deficits at follow-up [28]. Low plasma levels of the neurotrophic factor BDNF were associated with a higher risk of falciparum cerebral malaria, disability, or death in a similar pediatric cohort in Uganda [29]. Last, low plasma levels of NCAM-1, a protein involved in neural development and synaptic formation, were associated with severe malarial anemia in Malian children [30], a condition recently shown to also contribute to neurocognitive impairment [28]. Our findings of increased S100B, Tau, and UCH-L1 plasma levels and decreased BDNF and NCAM-1 during *Pk* infection align with these observations (Table 1), suggesting potential cerebral involvement, neuronal injury, BBB disruption, and loss of neuroprotective mechanisms.

**Table 1.** Brain Injury Biomarkers With Significant Group Differences: Comparison to Plasma Levels Reported by Other Studies

Biomarker	Malaysia Cohort		Other Studies						
	Control Group (n = 19 Adults)	*Pk*-Infected (n = 19 Adults)	Study [Reference]	Country	*Plasmodium* Species	Technique	Control Group	Case Group 1	Case Group 2
BDNF	1516.2 (835.5–2289.6)	146.2 (91.7–207.5)	McDonald et al, 2017 [29]	Uganda	*P falciparum*	ELISA	NA	1.8 (2.5)^a^ (n = 100 SNCM children)	1.1 (1.3)^a^ (n = 79 CM children)
GFAP^b^	78.1 (66.0–111.8)	29.9 (21.1–46.5)	Datta et al, 2023 [28]	Uganda	*P falciparum*	Simoa	100.6 (42.3) (n = 20 children aged ≥5 y)	69. 6 (56.5) (n = 30 SMA children aged ≥5 y)	86.8 (57.7) (n = 44 CM children aged ≥5 y)
S100B	27.3 (27.3–27.3)	1282.2 (544.1–2321.2)	Mohanty et al, 2022 [17]	India	*P falciparum*	Luminex	NA	617.9 (490.9) (n = 9 UM adults)	4121.9 (11 006.9) (n = 19 SNCM adults)
Tau total	0.6 (0.3–0.8)	3.7 (1.5–6.4)	Datta et al, 2021 [26]	Uganda	*P falciparum*	Simoa	2.9 (1.9) (n = 21 children aged ≥5 y)	4.9 (5.3) (n = 30 SMA children aged ≥5 y)	5.9 (3.7) (n = 45 CM children aged ≥5 y)
UCH-L1	49.3 (25.0–70.4)	336.6 (223.2–407.3)	Datta et al, 2023 [28]	Uganda	*P falciparum*	Simoa	9.7 (10.8) (n = 20 children aged ≥5 y)	29.9 (27.9) (n = 30 SMA children aged ≥5 y)	52.9 (78.3) (n = 44 CM children aged ≥5 y)

Data are median (interquartile range). Biomarker levels are expressed in picograms per milliliter unless stated otherwise. Children were defined as individuals aged ≤14 years.

Abbreviations: BDNF, brain-derived neurotrophic factor; CM, cerebral malaria; ELISA, enzyme-linked immunosorbent assay; GFAP, glial fibrillary acidic protein; NA, not applicable; *Pk*, *Plasmodium knowlesi*; SMA, severe malarial anemia; SNCM, severe noncerebral malaria; S100B, S100 calcium-binding protein β; UCH-L1, ubiquitin carboxy-terminal hydrolase L1; UM, uncomplicated malaria.

^a^Values are reported in nanograms per milliliter.

^b^Values out of range in our cohort: GFAP (n = 1 in the *Pk*-infected group).

In broader clinical contexts, biomarkers elevated in our *Pk*-infected cohort have been associated with diverse conditions and are used as indicators of brain injury or dysfunction. For instance, patients with Parkinson disease had significantly higher plasma levels of the synaptic vesicle trafficking regulator αSyn compared to healthy controls [31], and elevated levels of postsynaptic protein NRGN were associated with mild to acute traumatic brain injury [32]. The increased levels of αSyn and NRGN in our *Pk*-infected patients suggest potential neurological implications, prompting further investigation into the underlying mechanisms of knowlesi malaria pathogenesis and their clinical significance over time.

In our study, several biomarkers were significantly decreased in *Pk*-infected individuals compared to healthy controls, including GFAP, CaBD, CNTN1, and KLK6. The astrocytic intermediate filament protein GFAP, used clinically to assess traumatic brain injury [33], glymphatic function [34], and neurodegeneration [35], was not found to be elevated in Ugandan children with falciparum cerebral malaria [28]. In contrast, the observed differences in GFAP levels in our groups may suggest altered astroglial and/or glymphatic functions in infected participants, supporting our hypothesis of cerebral involvement in *Pk* malaria. CNTN1, crucial for neural development and synaptic formation, was decreased in our *Pk*-infected patients. Downregulated serum levels of this biomarker predicted cognitive and motor declines in patients with Parkinson disease [36], Last, plasma levels of the neuroinflammation modulator KLK6 were significantly increased in patients with advanced Alzheimer disease compared with healthy controls [37]. Although challenging to interpret, these results suggest cerebral involvement during *Pk* infection and a potential risk of cognitive decline, indicating a complex interplay between neuroinflammatory and neuroprotective mechanisms that warrant further investigation.

Despite our expectations based on existing malaria literature (Supplementary Table 1), we did not observe significant group differences in the iron-trafficking protein NGAL, a recognized biomarker of neuroinflammation [38]. Elevated plasma NGAL levels were associated with cerebral malaria in adult patients from India and distinguished between fatal and nonfatal outcomes [18]. Similarly, plasma NfL levels in Mozambican children with uncomplicated and severe falciparum malaria showed a significant increase over time, particularly in severe malaria cases with neurological symptoms, suggesting NfL as a potential follow-up biomarker of brain injury in malaria [39]. Last, the glycoprotein fetuin A was previously reported as elevated in serum from Malaysian patients with *Pk* malaria compared to uninfected controls [40]. Our findings, diverging from anticipated outcomes, underscore the complexity of biomarker dynamics in infections with different *Plasmodium* species and emphasize the need for further exploration in the context of *Pk* malaria.

In our hierarchical clustering analysis, we observed distinct group profiles for brain injury biomarker levels, indicating differences between *Pk*-infected patients and healthy controls. Most infected individuals clustered together with elevated levels of specific biomarkers such as S100B, αSyn, Park7, and TDP-43, suggesting a cohesive pattern of cerebral injury. However, 2 infected individuals exhibited an atypical clustering with the healthy controls, indicating potential overlap or milder infection. Conversely, in the control group, several biomarkers including BDNF, GFAP, CaBD, and CNTN1 displayed higher levels and clustered together, representing a baseline biomarker profile in healthy individuals.

Clustering of vascular biomarkers revealed clear and distinctive group profiles. Uninfected individuals formed a cohesive cluster with high levels of PDGF-AA, Ang-1, PDGF-BB, Serpin E1, and BMP-9, revealing a healthy, baseline vascular profile. Conversely, the infected group cluster had high levels of VCAM-1 and Ang-2/Ang-1 ratio, indicative of vascular involvement and endothelial activation during *Pk* infection. The elevated Ang-2/Ang-1 ratio observed in our infected group aligns with findings from previous studies on severe malaria caused by other *Plasmodium* species [41, 42]. Similarly, increased levels of VCAM-1 have been reported in both falciparum and vivax malaria infections [43, 44].

Last, total IgG antibody responses observed against *Plasmodium* spp antigens, including *Pk*, were low not only among most uninfected controls but also *Pk-*infected patients (Supplementary Figure 6), and did not form distinct clusters between the 2 groups (Supplementary Figure 7). This suggests the cohort was largely malaria naive, with low reactivity to long-term infection markers.

In our cohort, no correlations were found between participant’s age or parasitemia and circulating levels of brain injury biomarkers (Supplementary Figures 4–5). Research in primate models have contributed valuable data on pathophysiology of *Pk* infection, showing that *Macaca fascicularis* monkeys typically control parasitemia and develop chronic infections without severe brain injury [45]. In humans, however, our findings are in line with previous evidence of pathological mechanisms similar to the ones seen in severe falciparum malaria [7], including brain sequestration [13, 16], as well as microvascular and neuronal injury [25, 42]. We hypothesize that neuropathology in *Pk* infection may involve similar mechanisms to falciparum but that lower parasitemia and distinct host responses could modify the extent and nature of brain damage, explaining the absence of coma. In addition, the reported sequestration of *Pk*-infected erythrocytes [13, 16] could lead to a hidden biomass not reflected by parasitemia, as described in falciparum malaria [46].

Our study has several limitations. First, due to the initial scope of the parent study [23], detailed clinical data on neurological complications and renal failure among the *Pk*-infected group were not collected, restricting our ability to fully contextualize the observed alterations in brain injury biomarkers (Supplementary Table 6). As a result, we were unable to investigate direct associations between elevated brain injury biomarkers in *Pk* infection and brain function alterations, as described in severe falciparum malaria [17, 18]. Similarly, recent studies link AKI to brain injury pathogenesis [17] and long-term neurocognitive sequelae [8, 9], but this assessment was not possible in our cohort. However, AKI is a common complication of knowlesi malaria [21], and it is plausible that similar distant organ pathways are involved, resulting in an exacerbation of the brain changes [20]. Further work is warranted to explore this hypothesis. Second, without follow-up samples from the parent study, we were not able to evaluate whether the elevated biomarker levels return to baseline or increase over time. This would be particularly relevant for NfL, which typically increases after cerebral insult, and would provide insight into the resolution or persistence of brain injury following *Pk* infection [27, 39]. Last, a symptomatic, nonmalaria control group would have allowed stronger ascertainment of associations between biomarkers and *Pk* infection.

In conclusion, our study represents the first comprehensive assessment of surrogate markers of cerebral involvement in *Pk*-infected patients from Malaysia. Despite the limitations of this exploratory analysis, our findings indicate that *Pk* infection may impact brain and vascular health through pathways similar to the ones described for *P falciparum*, leading to elevated levels of brain injury, inflammation, and vascular biomarkers compared to healthy controls. Measuring the same panel of biomarkers longitudinally in well-characterized cohorts of patients with severe falciparum and severe vivax malaria is warranted to allow further comparative pathogenesis analyses during infection with *Pk* and other *Plasmodium* spp. Our study sets the stage for further research into pathophysiology and long-term brain impact of *Pk* malaria through follow-up neuroimaging and neurocognitive evaluations.

## Supplementary Material

jiae553_Supplementary_Data

## Supplementary Data

Supplementary materials are available at *The Journal of Infectious Diseases* online (http://jid.oxfordjournals.org/). Supplementary materials consist of data provided by the author that are published to benefit the reader. The posted materials are not copyedited. The contents of all supplementary data are the sole responsibility of the authors. Questions or messages regarding errors should be addressed to the author.

## Notes

***Acknowledgments.*** We extend our gratitude to the local health officers and personnel from Mersing, Kota Tinggi, and Pejabat Kesihatan Dareah Kota Tinggi (Johor), Kuala Lipis and Kesihatan Betau (Pahang), Sungai Siput (Perak), Kuala Kubu Bharu (Selangor), and Setiu (Trengganu) for their invaluable assistance with sampling. We express our sincere appreciation to Dr Christian Bottomley, Ana Chopo-Pizarro, and Helena Brazal Monzó at the London School of Hygiene and Tropical Medicine (LSHTM) for their invaluable insights. Last, we thank Dr Dibyadyuti Datta and Dr Andrea Conroy for their help compiling the data shown in Table 1, which greatly enhanced the quality of this manuscript.

***Author contributions*.** C. B.-C.: Conceptualization, methodology, investigation, formal analysis and interpretation, visualization, writing–original draft, writing–review and editing. E. D.: Methodology, formal analysis and interpretation, visualization, writing–review and editing. N. R. N. and M.-Y. L.: Conceptualization, methodology, investigation, writing–review and editing. W. S., K. K. A. T., and C. D.: Methodology, investigation, resources, writing–review and editing. S. K.: Conceptualization, investigation, writing–review and editing. Y.-L. L. and S. C. W.: Conceptualization, methodology, investigation, resources, supervision, writing–review and editing. All authors approved the final version.

***Ethics approval.*** This study has been reviewed and approved by the Medical Research and Ethics Committee of the Ministry of Health in Malaysia (reference NMRR ID 22-02557-1KV) and by the Observational/Interventions Research Ethics Committee at LSHTM (reference 27902). The shipment of samples from Malaysia to the UK was performed in compliance with national and international regulations in both sites. The samples are registered with the Human Tissue Authority, in accordance with UK national guidelines.

***Financial support.*** S. C. W. is supported by the Medical Research Council UK (award number MR/S009450/1) and the National Institute of Allergy and Infectious Diseases of the National Institutes of Health (award number U19AI089676).
