# Supplementary material for: Plasmodium knowlesi Infection Is Associated With Elevated Circulating Biomarkers of Brain Injury and Endothelial Activation
Source: J Infect Dis. 2024 Dec 10;231(5):e966–75. doi: 10.1093/infdis/jiae553 (PMC12128082; doi:10.1093/infdis/jiae553)
Supplement: jiae553_Supplementary_Data [file jiae553_supplementary_data.pdf]

SUPPLEMENTARY MATERIALS

Plasmodium knowlesi infection is associated with elevated circulating biomarkers of brain injury and endothelial activation

*Cesc Bertran-Cobo[1,2,3 ϕ], Elin Dumont[1 ϕ], Naqib Rafieqin Noordin[4], Meng-Yee Lai[4], William Stone[1], Kevin KA Tetteh[1Ψ], Chris Drakeley[1], Sanjeev Krishna[4,5,6,7], Yee-Ling Lau[4 † *], Samuel C Wassmer[1 † *]*

Affiliations:

[1]Department of Infection Biology, London School of Hygiene and Tropical Medicine, London, UK

[2]Department of Psychiatry and Mental Health, University of Cape Town, Cape Town, South Africa

[3]Neuroscience Institute, University of Cape Town, Cape Town, South Africa

[4]Department of Parasitology, Faculty of Medicine, Universiti Malaya, Kuala Lumpur, Malaysia

[5]Institut Für Tropenmedizin, Eberhard Karls Universität Tübingen, Tübingen, Germany

[6]Centre de Recherches Médicales de Lambaréné (CERMEL), Lambaréné, Gabon

[7]Clinical Academic Group in Institute for Infection & Immunity, St George's University of London, London, UK

[Ψ] Current affiliation: FIND, Campus Biotech, Chemin des Mines 9, 1202, Geneva, Switzerland

[ϕ] These authors share first authorship.

[†] These authors share last authorship.

***Correspondence:**

Yee-Ling Lau, Universiti Malaya, Kuala Lumpur, Malaysia: lauyeeling@um.edu.my

Sam C Wassmer, London School of Hygiene & Tropical Medicine, London, UK: sam.wassmer@lshtm.ac.uk

Table of contents

Supplementary Methods
Sample collection procedures 3
Biomarker panel selection 3
Immunoseroprevalence assay 3

Supplementary Results
Biomarkers with values out of detection range 4
Hierarchical clustering of immune biomarker levels 4
Correlation of biomarker levels with age and parasitemia 4
Serological markers of previous malaria exposure 5

Supplementary Figures
Supplementary Figure 1. Levels of blood circulating immune/inflammatory biomarkers 6
Supplementary Figure 2. Levels of blood circulating vascular biomarkers 8
Supplementary Figure 3. Correlation matrixes 9
Supplementary Figure 4. Biomarker levels: correlation with age 11
Supplementary Figure 5. Biomarker levels: correlation with parasitaemia 13
Supplementary Figure 6. Immunoseroprevalence assay: All markers 15
Supplementary Figure 7. Immunoseroprevalence assay: Hierarchical clustering 16

Supplementary Tables
Supplementary Table 1. Plasma biomarkers of brain alterations or cerebral injury 17
Supplementary Table 2. Plasma biomarkers of infection and immune activation 21
Supplementary Table 3. Vascular biomarkers 26
Supplementary Table 4. List of antigen targets utilised in the immunoassay to assess malaria exposure ... 29
Supplementary Table 5. Individual biomarker group comparisons (Wilcoxon test) 30
Supplementary Table 6. Clinical data extracted from the parent study 33

References 35

1 Supplementary methods

1.1 Sample collection procedures

Samples were acquired using EDTA anti-coagulant tubes. Informed consent was obtained prior to sample collection. All participants were allowed sufficient time to consider their participation in the project. Individual data were collected and recorded anonymously. *Plasmodium* infection and speciation in each collected sample were confirmed by microscopic examination of Giemsa-stained blood smears and nested polymerase chain reaction (PCR) based on the 18S rRNA gene. Out of the 50 individuals sampled as part of the parent project, 38 had associated clinical information needed for this study, including parasitemia.

1.2 Biomarker panel selection

Our literature review-based selection followed the assumption that any plasma biomarker ever reported to be altered in any species of human malaria could potentially be altered in our cohort of Pk patients. For brain injury biomarkers, a second premise applied: since cerebral malaria can cause long-lasting cognitive disorders, any plasma marker reported to be increased or decreased in cognitively impaired subjects could also be altered in our patients. To inform the biomarker panel selection, a systematic search of scientific literature on biomarker plasma levels in human patients was conducted in PubMed database, including all relevant publications from 2010 onwards.

1.3 Immunoseroprevalence assay

Sera were screened against a previously optimised panel of 14 blood-stage antigens representing varied markers of malaria exposure, listed in **Supplementary Table 3**. This incorporated 6 *Pk* antigens: *Pk*AMA1 and *Pk*MSP1$_{19}$, historical (long-term) exposure markers that can persist in blood for several years with repeated infections, PkSera3 Ag2 and PkSSP2/TRAP, which have previously been utilised as markers in seroprevalence studies on Malaysian populations (1–3), and *Pk*1 and *Pk*8, exploratory antigens that demonstrated high immunogenicity in preliminary data from assay screenings of pooled sera from *Pk*-infected Malaysian hyperimmune individuals (**K. Tetteh**, **personal communication**). For *P. falciparum* and *P. vivax*, markers of historical exposure, *Pf* / *Pv*MSP1$_{19}$ and *Pf* / *Pv*AMA1, and markers of recent (short-term) exposure known to persist in blood for up to 6-12 months following infection, *Pf*Etramp5 Ag1 and *Pv*RBP 2b, were included (4,5). *Pm*MSP1$_{19}$ and *Po*MSP1$_{19}$ were used to assess historical exposure to *P. malariae* and *P. ovale*, respectively. Additionally, tetanus toxoid vaccine protein from *Clostridium tetani* and glutathione-S-transferase (GST) from *Schistosoma japonicum* were included as non-malaria internal assay controls (6).

The Luminex assay was performed as previously described (7). Briefly, based on previously identified antigen-specific optimal EC_{50} concentrations, each antigen was covalently coupled to a colour-coded MagPlex® bead region (MagPlex, Luminex Corp., Austin, TX) via N-Hydroxysuccinimide/1-Ethyl-3-(3-dimethylaminopropyl)carbodiimide (NHS/EDC) chemistry. Test sera were prepared at 1/400 in diluent buffer (1x Phosphate Buffer Saline (PBS); 0.05% Tween® 20; 0.5% bovine serum albumin; 0.02% sodium azide; 0.1% casein; 0.5% polyvinyl alcohol; 0.5% polyvinylpyrrolidone; 15.25ug/mL *E. coli* lysate) and incubated at 4°C overnight. Antigen-coupled beads were incubated with 50µL of diluted samples and incubated for 90 minutes at room temperature with shaking at 600 RPM before incubation with 50µL of 1/200 R-Phycoerythrin-

conjugated AffiniPure $F(ab')_2$ Fragment Goat Anti-Human IgG secondary antibody (JacksonImmunoResearch; 109-116-098) for 90 minutes under the same conditions (38).

Pooled sera from hyperimmune individuals in Malaysia (*Pk*+), Tanzania (CP3), and Peru (S1) were included as *Pk, P. falciparum, and P. vivax* positive controls, respectively, in 6-point 5-fold serial dilution curves (1/10 - 1/31250). Commercial WHO reference reagents for anti-*P. falciparum* (10/198) human serum and anti-*P. vivax* (19/198) human plasma were also added as positive controls at 1/400 and 1/4000. Public Health England (PHE) malaria naïve human sera (n=30) were included as negative controls at 1/400. Two wells of diluent buffer served as blank controls to allow subtraction of background signal. The plates were read using the MAGPIX© instrument (Luminex Corp., Austin, TX) with the raw data recorded as Median Fluorescent Intensity (MFI) values using an acquisition target of ≥30 beads per region per well. The data were background-adjusted and normalised as described by Wu *et al* (7).

2 Supplementary Results

2.1 Biomarkers with values out of detection range

Since S100B and $A\beta_{(1-42)}$ plasma levels were below the range of detection in most uninfected subjects, we first compared the proportion of individuals with detectable levels of these biomarkers in each group using a Chi-square test with Yates' correction. The percentage of participants with plasma levels within the range of detection was significantly higher in the *Pk*-infected group (19/19, 100%) than in the uninfected control group (4/19, 21.05%) ($p<0.0001$). Similarly for $A\beta_{(1-42)}$, the proportion of individuals with detectable plasma levels in the *Pk*-infected group (17/19, 89.47%) was significantly higher compared with their uninfected peers (7/19, 36.84%) ($p= 0.0025$).

To enable group comparisons for these two biomarkers, participants with values below the range of detection were assigned a numerical value corresponding to half the value of the lower threshold of detection. According to the manufacturer's information, these values were 27.31 pg/mL for S100B and 0.22 pg/mL for $A\beta_{(1-42)}$. Achieved this, Wilcoxon tests revealed significantly higher plasma S100B levels in the *Pk*-infected group, which survived Bonferroni correction for multiple comparisons ($p<0.0001$), whereas no significant group differences were found for $A\beta_{(1-42)}$ plasma levels.

2.2 Hierarchical clustering of immune biomarker levels

Clustering of biomarkers associated with infection and immune activation did not reveal distinct separation between *Pk*-infected patients and healthy controls. Higher levels of IL-1RA and MPO predominantly clustered in the infected group (17/19, 89.47%), and two control individuals also exhibited this pattern (2/19, 10.53%). One infected individual (1/19, 5.26%) displayed very high levels of IL-1β, GM-CSF, TNF-α, CCL4, and CCL2. Another infected individual (1/19, 5.26%) exhibited very high levels of OPN and IL-10. Additionally, one control individual showed high levels of IL-6 (1/19, 5.26%).

2.3 Correlation of biomarker levels with age and parasitemia

No significant correlations were identified between the levels of any of the examined biomarkers and the age of participants within each respective group, as illustrated in **Supplementary Figure 4**. Furthermore,

within the *Pk*-infected group, no significant correlations were observed between biomarker levels and the percentage of parasitemia (**Supplementary Figure 5**).

2.4 Serological markers of previous malaria exposure

Overall, the majority of participants generated low antibody responses across *Pk, P. vivax, P. falciparum, P. ovale*, and *P. malariae* antigens with the exception of a few high responders (**Supplementary Figure 6**). Clustering of exposure markers in the hierarchical heatmap analysis did not reveal distinct separation between responses of *Pk*-infected patients and uninfected controls (**Supplementary Figure 7**). Among *Pk* antigens, uninfected controls exhibited higher responses against *Pk*8 ($p<0.0001$), *Pk*SERA3 Ag2 ($p=0.0406$), and *Pk*1 ($p=0.0250$) than *Pk*-infected patients, but no differences were observed for *Pk*AMA1 ($p>0.9999$), *Pk*MSP1$_{19}$ ($p=0.7778$), or *Pk*SSP2 ($p=0.2592$).

Interpreting these results, total IgG antibody responses observed against *Plasmodium* spp. antigens, including *Pk*, were low not only among most uninfected controls but also *Pk*-infected patients, and did not form distinct clusters between the two groups. This suggests the cohort was largely malaria naïve, with low reactivity to long-term infection markers *Pk*/*Pv*/*Pf*/*Pm*/*Po*MSP1$_{19}$ and *Pk*/*Pv*/*Pf*AMA1 observed at levels comparable to the PHE malaria naïve controls in all but two *Pk*-infected patients (8). As naturally acquired responses against these antigens develop cumulatively with repeated exposure, this indicates the occurrence of very few historical *Plasmodium* infections across participants (9). This could highlight a lack of clinical immunity, rendering *Pk*-infected patients more susceptible to potential malaria complications reflected in the brain and vascular biomarker profiles in this study, though this was not possible to investigate due to insufficient clinical information. The lack of elevated responses to *Pk* antigens SSP2, SERA3 Ag2, *Pk*1 and *Pk*8 may indicate there was inadequate time for *Pk*-infected patients to mount a response prior to the sampling period (1).

Among uninfected controls, serological analyses identified two individuals exhibiting notably higher responses: one control to *Pk* exploratory marker *Pk*8 and *P. vivax* short-term exposure marker *Pv*RBP2b, and another also to *Pv*RBP2b. Although this suggests previous exposures to *Pk* and *P. vivax*, this did not appear to explain any atypical deviations in plasma biomarker levels observed in a minority of individuals in this group.

3 Supplementary Figure 1

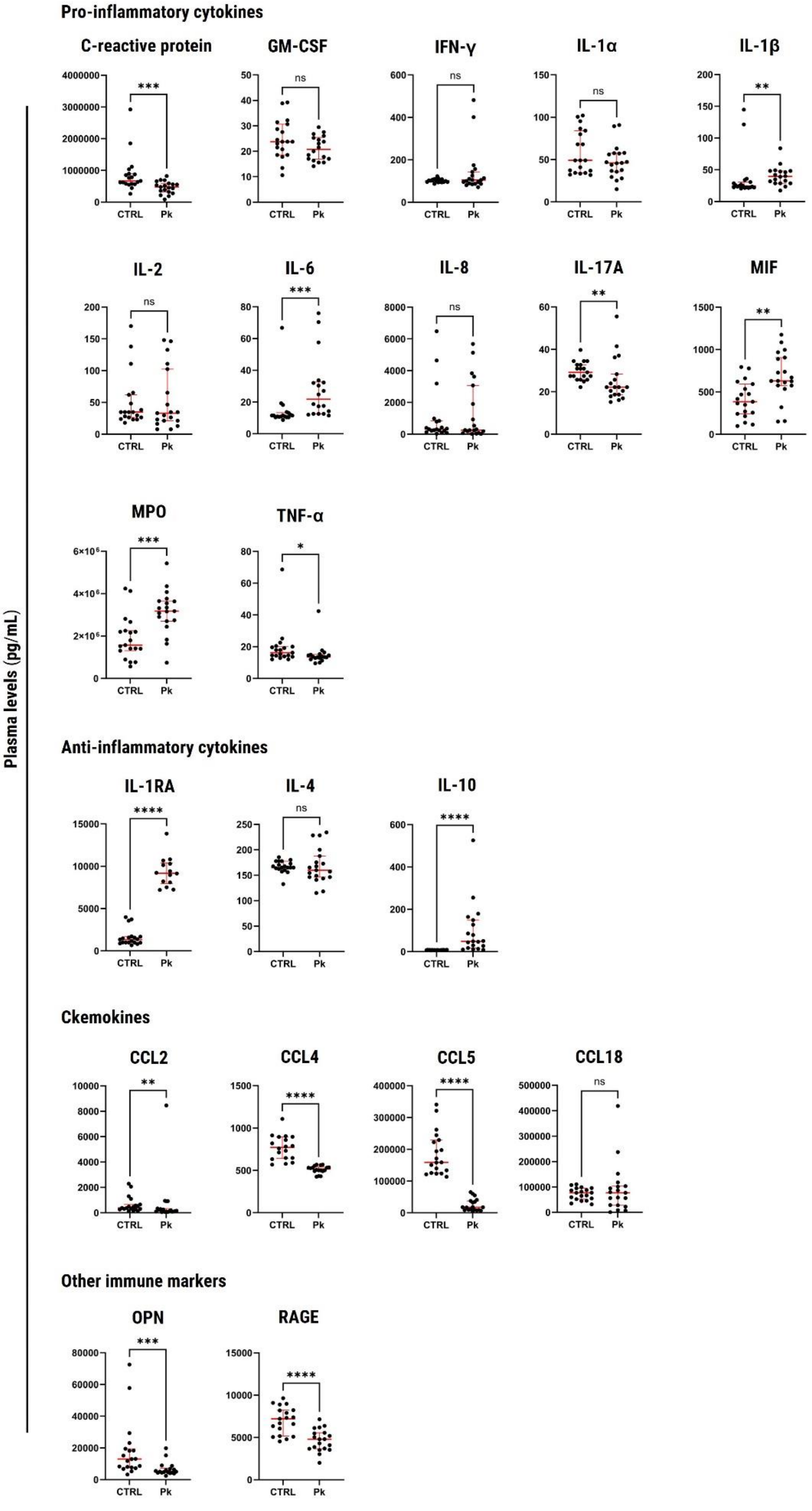

Supplementary Figure 1. Group comparisons: Levels of blood circulating immune/inflammatory biomarkers. Dot plots represent individual data points. Median and interquartile range (IQR) are indicated by horizontal lines and error bars in red, respectively.

CCL2: chemokine (C-C motif) ligand 2; **CCL4**: chemokine (C-C motif) ligands 4; **CCL5**: chemokine (C-C motif) ligand 5; **CCL18**: chemokine (C-C motif) ligand 18; **CNS**: central nervous system; **CRP**: C-reactive protein; **CSF**: cerebrospinal fluid; **GM-CSF**: granulocyte-macrophage colony-stimulating factor; **IFN-γ**: interferon gamma; **IL-1α**: interleukin 1 alpha; **IL-1β**: interleukin 1 beta; **IL-1RA**: interleukin 1RA; **IL-2**: interleukin 2; **IL-4**: interleukin 4; **IL-6**: interleukin 6; **IL-8**: interleukin 8; **IL-10**: interleukin 10; **IL-17A**: interleukin 17; **ILC**: innate lymphoid cells; **MIF**: migration inhibitory factor; **MPO**: myeloperoxidase; **NK**: natural killer; **OPN**: osteopontin; **RAGE**: receptor for advanced glycation end-products; **TNF-α**: tumour Necrosis Factor alpha; **VSMC**: vascular smooth muscle cells. **CTRL**: Control group; **Pk**: *Pk*-infected patients.

Wilcoxon test results: *p<0.05; **p<0.01; ***p<0.001; ****p<0.0001; ns: no significance.

4 Supplementary Figure 2

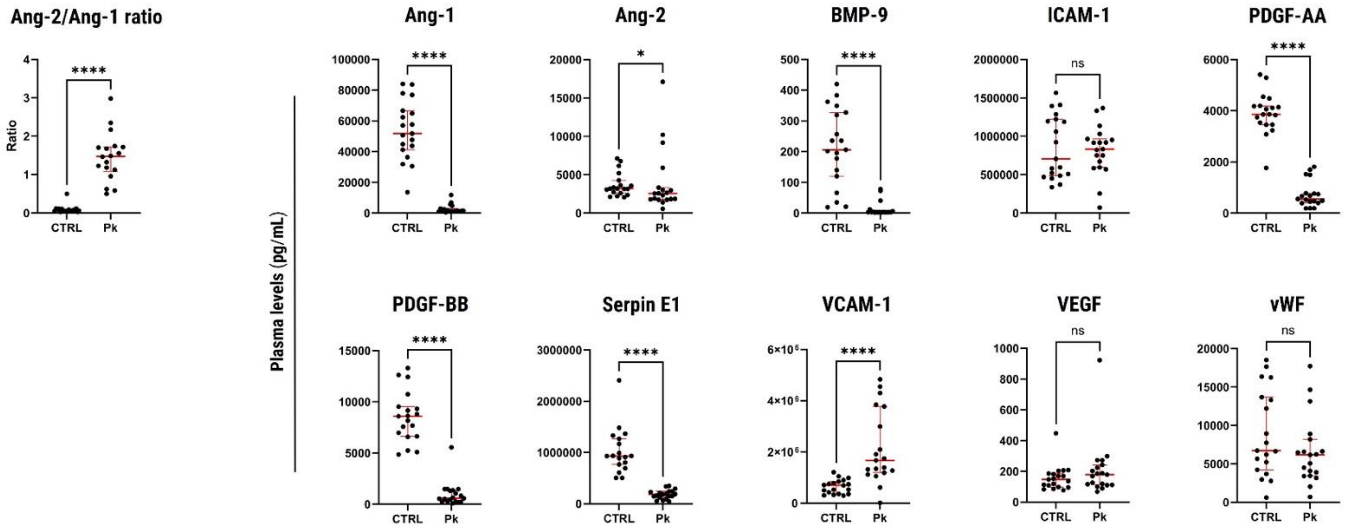

Supplementary Figure 2. Group comparisons: Levels of blood circulating vascular biomarkers. Dot plots represent individual data points. Median and interquartile range (IQR) are indicated by horizontal lines and error bars in red, respectively.

Ang-1: angiopoietin-1; **Ang-2**: angiopoietin-2; **Ang-2/Ang-1**: ratio between Ang-2 and Ang-1; **BMP-9**: bone morphogenetic protein 9; **ICAM-1**: intercellular adhesion molecule 1; **PDGF-AA**: platelet-derived growth factor AA; **PDGF-BB**: platelet-derived growth factor BB; **VCAM-1**: vascular cell adhesion molecule; **Serpin E1**: Serine Proteinase Inhibitor-clade E1; **PEDF**: pigment epithelium derived factor; **VEGF**: vascular endothelial growth factor; **vWF-A2**: von Willebrand Factor (A2 domain).

CTRL: Control group; **Pk**: *Pk*-infected patients.

Wilcoxon test results: *p<0.05; **p<0.01; ***p<0.001; ****p<0.0001; ns: no significance.

5 Supplementary Figure 3

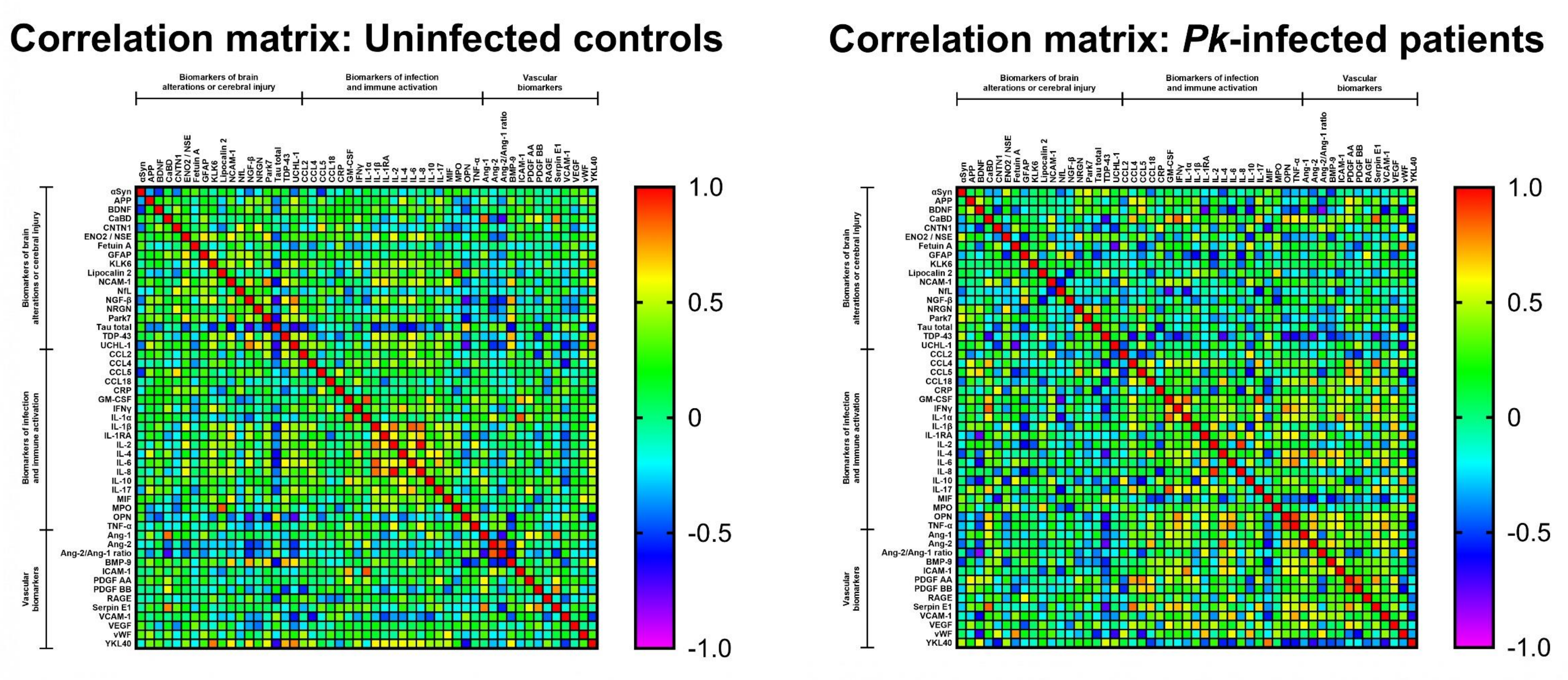

Supplementary Figure 3. Correlation matrices of biomarkers in healthy controls and *Pk*-infected patients. Correlation matrices displaying the relationships between brain injury, immune/inflammatory, and vascular biomarkers. The left panel shows the correlation matrix for the control group, and the right panel shows the correlation matrix for *Pk*-infected patients.

αSyn: alpha-Synuclein; **APP**: amyloid-beta precursor protein; **A**$\boldsymbol{\beta_{1\text{-}42}}$: amyloid beta (1-42); **BDNF**: brain-derived neurotrophic factor; **CaBD**: Calbindin D; **CNTN1**: contactin-1; **CSF**: cerebrospinal fluid; **ENO2** / **NSE**: Enolase 2 / Neuron-specific Enolase; **GFAP**: glial fibrillary acidic protein; **KLK6**: kallikrein 6 / neurosin; **NCAM-1**: neural cell adhesion molecule; **Lipocalin-2**: neutrophil gelatinase-associated lipocalin; **NGF-β**: nerve growth factor beta; **NfL**: neurofilament light chain; **NRGN**: neurogranin; **Park7**: Parkinsonism-associated deglycase; **S100B**: S100 calcium-binding protein β; **TDP-43**: TAR DNA-binding protein 43; **Tau**: total Tau protein; **UCH-L1**: ubiquitin carboxy-terminal hydrolase L1; **YKL40**: Chitinase-3-like protein 1.

CCL2: chemokine (C-C motif) ligand 2; **CCL4**: chemokine (C-C motif) ligands 4; **CCL5**: chemokine (C-C motif) ligand 5; **CCL18**: chemokine (C-C motif) ligand 18; **CRP**: C-reactive protein; **GM-CSF**: granulocyte-macrophage colony-stimulating factor; **IFN-γ**: interferon gamma; **IL-1α**: interleukin 1 alpha; **IL-1β**: interleukin 1 beta; **IL-1RA**: interleukin 1RA; **IL-2**: interleukin 2; **IL-4**: interleukin 4; **IL-6**: interleukin 6; **IL-8**: interleukin 8; **IL-10**: interleukin 10; **IL-17A**: interleukin 17; **MIF**: migration inhibitory factor; **MPO**: myeloperoxidase; **OPN**: osteopontin; **RAGE**: receptor for advanced glycation end-products; **TNF-α**: tumour Necrosis Factor alpha.

Ang-1: angiopoietin-1; **Ang-2**: angiopoietin-2; **Ang-2/Ang-1**: ratio between Ang-2 and Ang-1; **BMP-9**: bone morphogenetic protein 9; **ICAM-1**: intercellular adhesion molecule 1; **PDGF-AA**: platelet-derived growth factor AA; **PDGF-BB**: platelet-derived growth factor BB; **Serpin E1**: Serine Proteinase Inhibitor-clade E1; **VCAM-1**: vascular cell adhesion molecule; **VEGF**: vascular endothelial growth factor; **vWF-A2**: von Willebrand Factor (A2 domain). ***Pk***: *Plasmodium knowlesi*.

6 Supplementary Figure 4

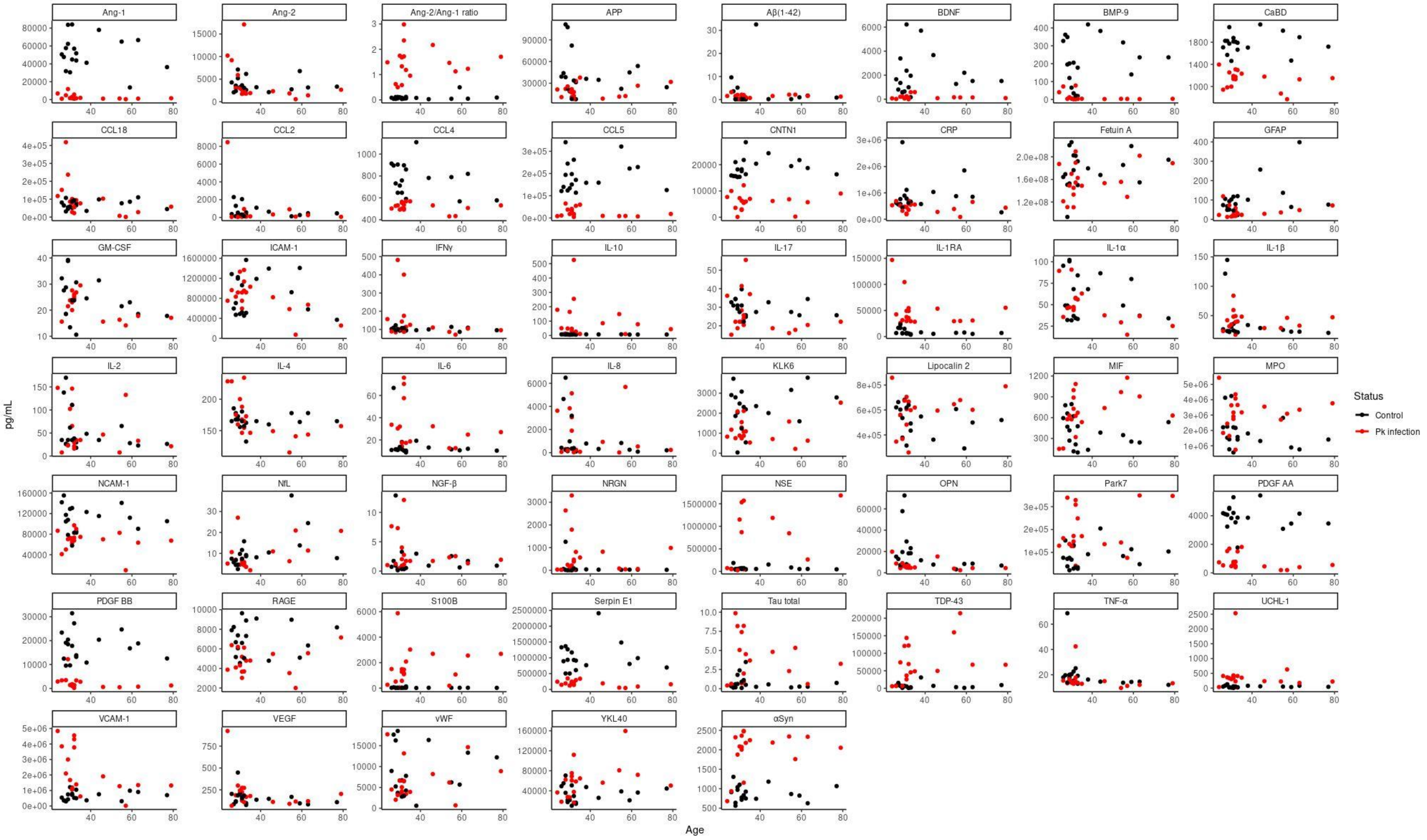

Supplementary Figure 4. Correlation plots of biomarker levels with participant's age. Scatter plots depicting the correlations between levels of all biomarkers and participant's age, colour-coded to distinguish between *Pk*-infected patients (red) and healthy controls (black) groups.

αSyn: alpha-Synuclein; **APP**: amyloid-beta precursor protein; **$A\beta_{1\text{-}42}$**: amyloid beta (1-42); **BDNF**: brain-derived neurotrophic factor; **CaBD**: Calbindin D; **CNTN1**: contactin-1; **CSF**: cerebrospinal fluid; **ENO2** / **NSE**: Enolase 2 / Neuron-specific Enolase; **GFAP**: glial fibrillary acidic protein; **KLK6**: kallikrein 6 / neurosin; **NCAM-1**: neural cell adhesion molecule; **Lipocalin-2**: neutrophil gelatinase-associated lipocalin; **NGF-β**: nerve growth factor beta; **NfL**: neurofilament light chain; **NRGN**: neurogranin; **Park7**: Parkinsonism-associated deglycase; **S100B**: S100 calcium-binding protein β; **TDP-43**: TAR DNA-binding protein 43; **Tau**: total Tau protein; **UCH-L1**: ubiquitin carboxy-terminal hydrolase L1; **YKL40**: Chitinase-3-like protein 1.

CCL2: chemokine (C-C motif) ligand 2; **CCL4**: chemokine (C-C motif) ligands 4; **CCL5**: chemokine (C-C motif) ligand 5; **CCL18**: chemokine (C-C motif) ligand 18; **CRP**: C-reactive protein; **GM-CSF**: granulocyte-macrophage colony-stimulating factor; **IFN-γ**: interferon gamma; **IL-1α**: interleukin 1 alpha; **IL-1β**: interleukin 1 beta; **IL-1RA**: interleukin 1RA; **IL-2**: interleukin 2; **IL-4**: interleukin 4; **IL-6**: interleukin 6; **IL-8**: interleukin 8; **IL-10**: interleukin 10; **IL-17A**: interleukin 17; **MIF**: migration inhibitory factor; **MPO**: myeloperoxidase; **OPN**: osteopontin; **RAGE**: receptor for advanced glycation end-products; **TNF-α**: tumour Necrosis Factor alpha.

Ang-1: angiopoietin-1; **Ang-2**: angiopoietin-2; **Ang-2/Ang-1**: ratio between Ang-2 and Ang-1; **BMP-9**: bone morphogenetic protein 9; **ICAM-1**: intercellular adhesion molecule 1; **PDGF-AA**: platelet-derived growth factor AA; **PDGF-BB**: platelet-derived growth factor BB; **Serpin E1**: Serine Proteinase Inhibitor-clade E1; **VCAM-1**: vascular cell adhesion molecule; **VEGF**: vascular endothelial growth factor; **vWF-A2**: von Willebrand Factor (A2 domain). ***Pk***: *Plasmodium knowlesi*.

7 Supplementary Figure 5

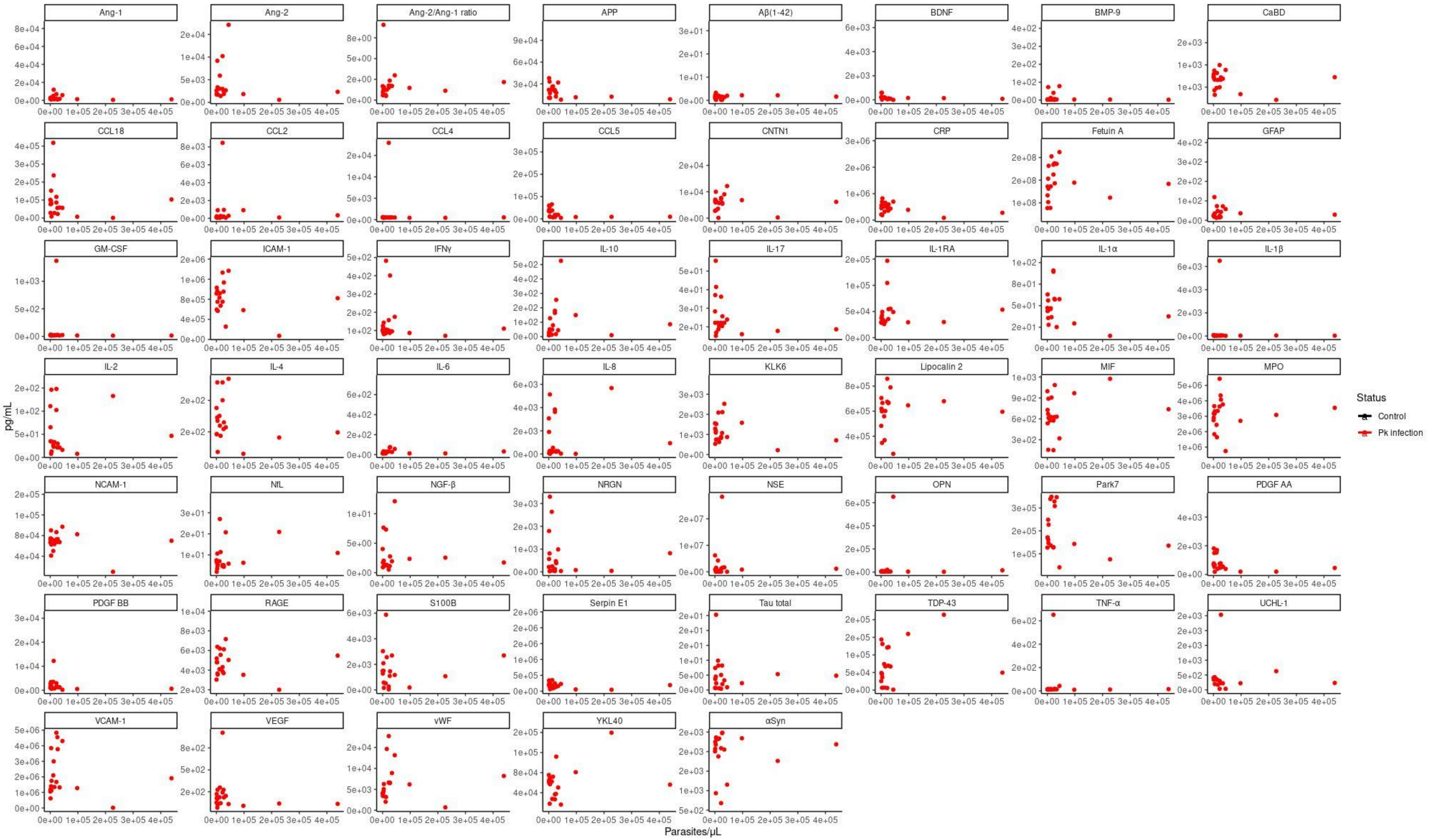

Supplementary Figure 5. Correlation plots of biomarker levels with parasitaemia. Scatter plots depicting the correlations between levels of all biomarkers and parasitaemia in the *Pk*-infected group.

αSyn: alpha-Synuclein; **APP**: amyloid-beta precursor protein; **Aβ_{1-42}**: amyloid beta (1-42); **BDNF**: brain-derived neurotrophic factor; **CaBD**: Calbindin D; **CNTN1**: contactin-1; **CSF**: cerebrospinal fluid; **ENO2** / **NSE**: Enolase 2 / Neuron-specific Enolase; **GFAP**: glial fibrillary acidic protein; **KLK6**: kallikrein 6 / neurosin; **NCAM-1**: neural cell adhesion molecule; **Lipocalin-2**: neutrophil gelatinase-associated lipocalin; **NGF-β**: nerve growth factor beta; **NfL**: neurofilament light chain; **NRGN**: neurogranin; **Park7**: Parkinsonism-associated deglycase; **S100B**: S100 calcium-binding protein β; **TDP-43**: TAR DNA-binding protein 43; **Tau**: total Tau protein; **UCH-L1**: ubiquitin carboxy-terminal hydrolase L1; **YKL40**: Chitinase-3-like protein 1.

CCL2: chemokine (C-C motif) ligand 2; **CCL4**: chemokine (C-C motif) ligands 4; **CCL5**: chemokine (C-C motif) ligand 5; **CCL18**: chemokine (C-C motif) ligand 18; **CRP**: C-reactive protein; **GM-CSF**: granulocyte-macrophage colony-stimulating factor; **IFN-γ**: interferon gamma; **IL-1α**: interleukin 1 alpha; **IL-1β**: interleukin 1 beta; **IL-1RA**: interleukin 1RA; **IL-2**: interleukin 2; **IL-4**: interleukin 4; **IL-6**: interleukin 6; **IL-8**: interleukin 8; **IL-10**: interleukin 10; **IL-17A**: interleukin 17; **MIF**: migration inhibitory factor; **MPO**: myeloperoxidase; **OPN**: osteopontin; **RAGE**: receptor for advanced glycation end-products; **TNF-α**: tumour Necrosis Factor alpha.

Ang-1: angiopoietin-1; **Ang-2**: angiopoietin-2; **Ang-2/Ang-1**: ratio between Ang-2 and Ang-1; **BMP-9**: bone morphogenetic protein 9; **ICAM-1**: intercellular adhesion molecule 1; **PDGF-AA**: platelet-derived growth factor AA; **PDGF-BB**: platelet-derived growth factor BB; **Serpin E1**: Serine Proteinase Inhibitor-clade E1; **VCAM-1**: vascular cell adhesion molecule; **VEGF**: vascular endothelial growth factor; **vWF-A2**: von Willebrand Factor (A2 domain).

Pk: *Plasmodium knowlesi*.

8 Supplementary Figure 6

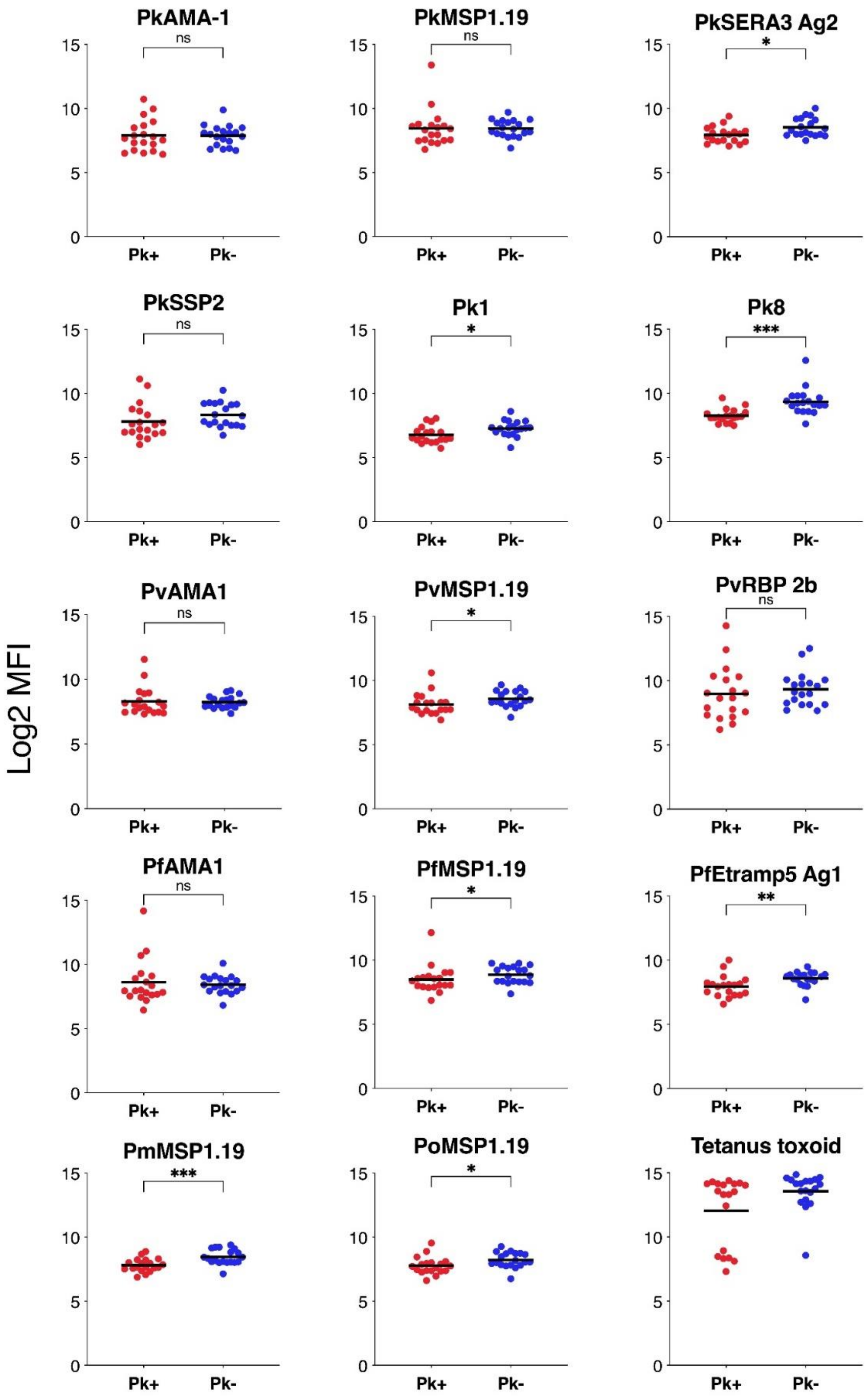

Supplementary Figure 6. Immunoseroprevalence assay: All markers. Dot plots illustrating participant antibody levels against markers (antigens) indicative of recent and historical exposure to *Plasmodium knowlesi* (*Pk*), *P. falciparum* (*Pf*), *P. vivax* (*Pv*), *P. malariae* (*Pm*), and *P. ovale* (*Po*) malaria.

9 Supplementary Figure 7

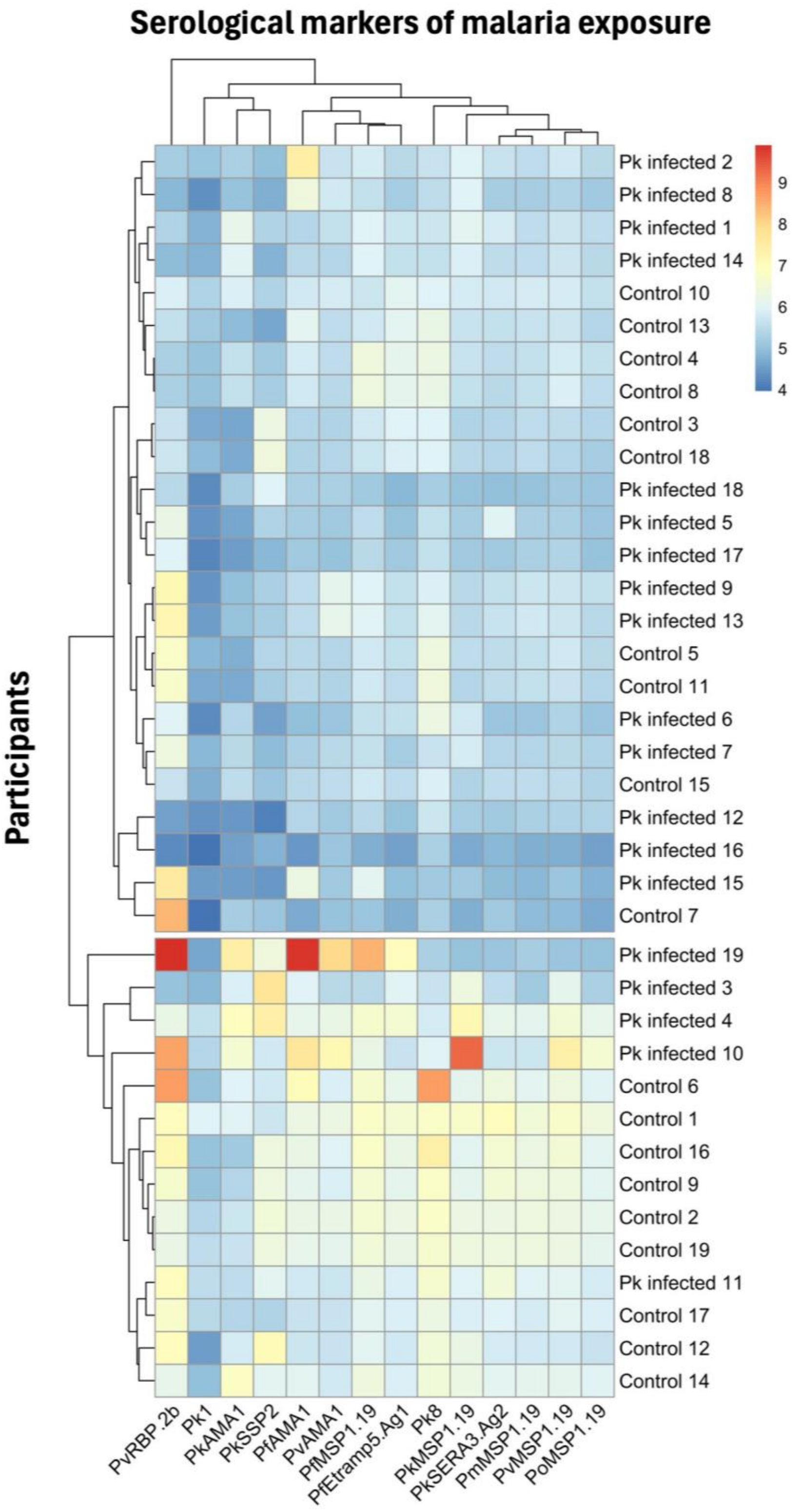

Supplementary Figure 7. Immunoseroprevalence assay: Hierarchical clustering. Hierarchical clustering analysis of antibody levels against malaria antigens in all participants. *Plasmodium knowlesi* (***Pk***), *P. falciparum* (***Pf***), *P. vivax* (***Pv***), *P. malariae* (***Pm***), *P. ovale* (***Po***).

10 Supplementary Table 1

Plasma biomarkers of brain alterations or cerebral injury		
**Marker**	**Description**	**Clinical significance**
**αSyn**	Neuronal intracellular protein. Regulates synaptic vesicle trafficking and neurotransmitter release. Its aggregation in the brain is associated with neurodegeneration.	Plasma αSyn levels were higher in Parkinson's Disease patients than healthy controls and patients with other neurodegenerative diseases (10,11).
**APP**	Neuronal transmembrane protein. Involved in the generation of synapses and axons, neurite growth, and neuronal adhesion. Undergoes proteolytic processes to generate **Aβ**.	In Japanese and Australian cohorts encompassing cognitively normal individuals, subjects with mild cognitive impairment, and Alzheimer's Disease patients, plasma APP/Aβ ratio predicted individual brain Aβ status as determined by PET imaging (12).
**$A\beta_{1-42}$**	APP-derived peptide. The imbalance between Aβ production and clearance, its misfolding, and accumulation in the extracellular space, are early factors in Alzheimer's disease.	Lower $A\beta_{1-42}$ plasma levels were associated with Aβ deposition in the brain of patients with varied degrees of cognitive decline, compared with other groups (13).
**BDNF**	Neurotrophic factor. Major regulator of synaptic transmission and plasticity in adult synapses. Homeostatic regulator of intrinsic neuronal excitability.	Plasma levels were significantly higher in Ugandan children with falciparum cerebral malaria compared with severe non-cerebral malaria patients. Plasma concentration increases were associated with resolution of severe malaria in this study (14).
**CaBD**	Cytosolic protein. Binds calcium with high affinity and regulates its availability in the cytoplasm, playing a role in transepithelial calcium transport.	Increased CSF levels could predict risk of future dementia in US patients who were cognitively normal at the time of recruitment (15).
**CNTN1**	Membrane protein, participates in nervous system development and neuronal-glia interactions in myelinated nerves.	Compared to healthy patients, downregulated CNTN1 serum levels predicted cognitive and motor declines in German patients with Parkinson's Disease (16).
**ENO2 / NSE**	Dimeric isoform of enolase, an enzyme involved in the metabolic process of glycolysis, relatively specific to neurons.	Plasma levels are significantly higher in patients with traumatic brain injury, compared with healthy controls (17–19).
**Fetuin A**	Serum glycoprotein, produced by hepatocytes, adipocytes, and choroid plexus cells. Involved in brain development, endocytosis, and bone tissue formation.	Compared with healthy controls, serum levels were significantly higher in Malaysian patients with knowlesi malaria, as revealed by two-dimensional electrophoresis and mass spectrometry analysis (20).
**GFAP**	Intermediate filament protein, specific to astrocytes. Involved in cell-cell communication and blood brain barrier functioning.	Used in clinical settings to determine injury severity and case management after traumatic brain injury (17,21).
		In Norwegian patients with CSF disorders, higher plasma GFAP concentrations were associated with impaired glymphatic function (22).

		In Chinese and UK cohorts, plasma GFAP level ranges distinguished patients with different neurodegenerative diseases (23,24).
**KLK6**	Serine protease, also known as neurosin. Participates in degradation processes against proteins such as APP or αSyn.	Plasma levels were significantly increased in Swedish patients with advanced Alzheimer's Disease, compared with healthy controls (25).
**NCAM-1**	Cell surface glycoprotein, expressed in neurons and glia. Involved in cell-cell adhesion, neurite outgrowth, synaptic plasticity, learning, and memory.	Longitudinal plasma NCAM-1 levels were among biomarker trajectories associated with falciparum-infected children in Mali, as identified by quantitative proteomics (26).
**NGAL**	Iron-trafficking protein. Participates in innate immunity by binding iron and impeding its uptake by bacteria, which limits their growth. Also plays a role in renal development.	Compared with uncomplicated cases, plasma levels were significantly higher in Indian adults with *falciparum* cerebral malaria and discriminated between fatal and nonfatal outcomes (27).
		Compared with non-dementia controls, plasma levels are significantly higher in patients with Alzheimer's Disease (28).
**NGF-β**	Neurotrophic factor. Regulates proliferation, differentiation, and survival of sympathetic and sensory neurons.	Compared with healthy controls, plasma levels are lower in patients with major depressive disorder and in patients with schizophrenia (29,30).
**NfL**	Subunit of intermediate filament proteins, exclusive to neurons. Highly expressed in axons, also present in dendrites and neuronal bodies.	Plasma levels were significantly higher in Ugandan children with falciparum cerebral malaria and severe malarial anaemia, compared with asymptomatic community children. Elevated levels associated with worse cognitive outcomes and mortality in children with cerebral malaria (31).
		Longitudinal analysis in Mozambican children with uncomplicated and severe falciparum malaria revealed that while plasma levels were similar upon admission, they increased over time, particularly in severe malaria cases with neurological symptoms (32).
		Increased plasma levels are detected in patients with traumatic or vascular injury, neuroinflammation, and neurodegeneration (17,33,34).
**NRGN**	Postsynaptic neuronal protein, expressed primarily in dendritic spines. Involved in the protein kinase C signalling pathway, regulates calmodulin availability.	Compared with healthy newborns, plasma levels were significantly higher in Irish babies with neonatal encephalopathy, and they were inversely associated with neurodevelopmental cognitive, motor, and language scores (35).
		Serum concentrations were significantly higher in Turkish and US patients with mild and acute traumatic brain injury, respectively, when compared with healthy controls (36,37).

**Park7**	Enzyme found in many tissues and organs, including the brain. Has a protective role against oxidative stress and cell death.	Compared to those of healthy controls, lymphocytes from patients at risk of developing prodromal Parkinson's Disease showed a decrease in the expression of Park7 (38).
**S100β**	Neurotrophic factor. Promotes astrogliosis and axonal proliferation. Highly expressed in astrocytes, it is one of the most abundant soluble proteins in the brain.	Increased plasma levels serve as a blood biomarker of cerebral small vessel disease and intracranial injury (39–41).
		Plasma levels were increased in Indian patients with falciparum severe malaria, compared with uncomplicated cases (42).
**TDP-43**	RNA-binding protein. Regulates the processing of RNAs involved in neuronal survival, as well as mRNAs that encode proteins relevant for neurodegenerative diseases.	Serum levels are decreased in patients with certain neurodegenerative diseases, such as frontotemporal dementia (43) and increased in other pathologies, such as sporadic amyotrophic lateral sclerosis (44).
**Tau**	Microtubule-associated protein, used as a biomarker of neuronal injury. Promotes microtubule assembly and stability, involved in neuronal polarity. Forms insoluble filaments that accumulate as neurofibrillary tangles in Alzheimer's Disease.	Plasma levels were increased in Ugandan children with falciparum cerebral malaria or severe malarial anaemia, compared with healthy controls. Plasma levels were associated with mortality and persistent neurocognitive impairment in young children with cerebral malaria (45).
		In Ugandan children with falciparum cerebral malaria, elevated CSF levels were associated with increased disease severity, malaria retinopathy, acute kidney injury, prolonged coma duration, and persistence of neurologic deficits up to 2 years post-discharge (46,47).
**UCH-L1**	Ubiquitin-protein hydrolase. Processes ubiquitin precursors and ubiquitinated proteins. Associated with neurofibrillary tangles in Alzheimer's Disease.	Plasma levels were significantly higher in Ugandan children with falciparum cerebral malaria and severe malarial anaemia, compared with asymptomatic community children. Elevated levels were linked to blood-brain barrier dysfunction and neurodeficits over follow-up (31).
**YKL-40**	Glycoprotein secreted by macrophages and other inflammatory cells during differentiation.	In Ugandan children with falciparum malaria, plasma levels were significantly increased in severe malarial anaemia and cerebral malaria versus uncomplicated malaria. Among severe cases, admission plasma levels predicted mortality with high sensitivity and specificity (48).
		In Ugandan children with severe *falciparum* malaria, admission plasma levels were elevated in cases who subsequently died. Levels correlated with markers of inflammation and endothelial activation (49).

αSyn: alpha-Synuclein; **APP**: amyloid-beta precursor protein; **$A\beta_{1-42}$**: amyloid beta (1-42); **BDNF**: brain-derived neurotrophic factor; **CaBD**: Calbindin D; **CNS**: central nervous system; **CNTF**: ciliary neurotrophic factor; **CNTN1**: contactin-1; **CSF**: cerebrospinal fluid; **ENO2** / **NSE**: Enolase 2 / Neuron-specific Enolase; **AHSG**: alpha 2-HS glycoprotein; **FGF-21**: fibroblast growth factor 21; **GDNF**: glial cell line-derived neurotrophic factor; **GFAP**: glial fibrillary acidic protein; **KLK6**: kallikrein 6 / neurosin; **NCAM-1**: neural cell adhesion molecule; **NGAL**: neutrophil gelatinase-associated lipocalin (also known as Lipocalin-2); **NGF-β**: nerve growth factor beta; **NfL**: neurofilament light chain; **NRGN**: neurogranin; **Park7**: Parkinsonism-associated deglycase; **RNA**: ribonucleic acid; **S100β**: S100 calcium-binding protein β; **TDP-43**: TAR DNA-binding protein 43; **Tau**: total Tau protein; **Tau pT181**: phosphorylated Tau protein; **UCH-L1**: ubiquitin carboxy-terminal hydrolase L1; **YKL40**: Chitinase-3-like protein 1.

Biomarker descriptions are extracted from **UniProt** (www.uniprot.org, last accessed January 2024).

Legend: Clinical findings related to: 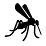 *Plasmodium falciparum* malaria infection; 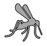 *Plasmodium knowlesi* malaria infection; 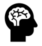 Neurological conditions.

11 Supplementary Table 2

Plasma biomarkers of infection and immune activation	
**Pro-inflammatory cytokines**	
**CRP**	In Ugandan children with falciparum cerebral malaria, plasma levels were significantly higher in retinopathy-positive patients compared with retinopathy-negative cases (46).
	Whole blood and plasma concentrations were significantly elevated in malaria-infected patients from Malaysia and Indonesian Papua, compared with healthy controls. Patients were mono-infected with *Plasmodium falciparum*, *vivax*, *knowlesi*, *malariae* or *ovale* as confirmed by PCR (50).
	In Cambodian asymptomatic participants, plasma levels were significantly higher in parasitaemic individuals compared with uninfected, age-, sex-, and village-matched controls. Patients had either a falciparum or vivax mono-infection, a *Plasmodium* infection with indeterminate species, or a mixed infection (51).
	In Brazilian patients with falciparum or vivax malaria, plasma levels were significantly higher in infected individuals compared with healthy controls. Levels were also higher in vivax patients than in *falciparum* cases (52).
**GM-CSF**	To the best of our knowledge, no studies have reported significant group differences in the context of human malaria.
**IFN-γ**	Plasma levels were significantly higher in Rwandan patients with severe malaria, compared with uncomplicated cases and controls (53).
	Plasma levels were significantly higher in Colombian patients with vivax severe malaria, compared with uncomplicated cases and healthy controls (54).
	In Brazilian patients with different forms of vivax malaria and controls, a network analysis revealed that IFN-γ, TNF-α, and CCL5 were crucial in the profile of mild malaria cases (55).
**IL-1α**	To the best of our knowledge, no studies have reported significant group differences in the context of human malaria.
**IL-1β***	A meta-analysis revealed that IL-1β blood levels were higher in severe malaria patients compared with uncomplicated cases. *Plasmodium spp*. was a confounder in the meta-analysis, showing no difference in IL-1β levels between falciparum-infected groups (56).
	In Indian patients with falciparum malaria, IL-1β levels were significantly the highest in severe cases with no brain injury, compared with other groups (57).
	In Beninese children with falciparum cerebral malaria, plasma levels were significantly higher in fatal cases compared with those who survived (58).
**IL-2**	Compared with Mozambican adults with life-long exposure to falciparum malaria, Spanish travellers diagnosed with malaria had significantly higher serum IL-2 levels (59,60).
**IL-6***	Plasma levels were significantly higher in Rwandan patients with severe malaria, compared with uncomplicated cases and controls (53).

		Plasma IL-6 levels were significantly higher in Colombian patients with vivax severe malaria, compared with uncomplicated cases and healthy controls (54).
		Plasma levels were increased in Brazilian patients with vivax malaria, compared with non-infected subjects with previous malaria episodes. Infected patients showed a strong correlation between CCL2 and IL-6 plasma levels, and moderate correlations between IL-6 and IL-10 (61).
		In Pakistani patients with vivax malaria, plasma levels were significantly higher in uncomplicated cases than healthy controls, and in complicated cases than in uncomplicated ones (62).
		Plasma levels were significantly higher in Malaysian patients with severe knowlesi malaria, compared with uncomplicated cases (63).
**IL-8**		In Beninese children with falciparum cerebral malaria, plasma levels were significantly higher in fatal cases compared with those who survived. In the former, IL-8 was identified as a risk factor for death by multivariate analysis (58).
**IL-17A**		Plasma levels were significantly higher in Ghanian children with severe falciparum malaria, compared with uncomplicated cases and non-malaria febrile controls (64).
		In Indian patients with falciparum malaria, plasma levels were higher in patients with multi-organ dysfunction compared with other severe malaria subgroups, suggesting IL-17 plays a role in renal inflammatory pathology during falciparum infection (65).
		Plasma levels were significantly higher in Rwandan patients with severe malaria, compared with uncomplicated cases and controls (53).
		Plasma levels were significantly higher in Brazilian patients with vivax malaria, compared with previously exposed, non-infected subjects and unexposed healthy donors (61).
**MIF**		Host MIF plasma concentrations positively correlated with vivax MIF levels in the plasma of Chinese patients with uncomplicated malaria (66).
**MPO**		In Cameroonian participants, plasma MPO levels were significantly higher in patients with falciparum malaria when compared with negative controls (67).
**TNF-α**		Serum levels were significantly higher in Indian patients with falciparum severe malaria and cerebral malaria, compared with healthy controls (68).
		In Beninese children with falciparum cerebral malaria, plasma levels were significantly higher in fatal cases compared with those who survived (58).
		TNF-α-producing monocytes were significantly lower in Malawian children with falciparum malaria compared with healthy controls. Cerebral malaria cases had the lowest values (69).
		Plasma levels were significantly higher in Rwandan patients with severe malaria, compared with uncomplicated cases and controls (53).
		In Brazilian patients with different forms of vivax malaria and controls, a network analysis revealed that IFN-γ, TNF-α, and CCL5 were crucial in the profile of mild malaria cases (55).

	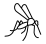	In Pakistani patients with vivax malaria, plasma levels were significantly higher in complicated cases compared with uncomplicated ones. TNF-α, IL-10, ICAM-1 and VCAM-1 were the best individual predictors of complicated *vivax* malaria (62).
**Anti-inflammatory cytokines**		
**IL-1RA**	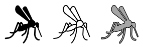	Most abundant cytokine measured in the serum of Malaysian Borneo patients with falciparum, vivax, or knowlesi malaria. Serum levels correlated with parasitaemia in subjects infected with any of the parasite species, and was associated with complications in knowlesi-infected patients (70).
**IL-4****	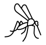	Plasma levels were significantly higher in Colombian patients with vivax severe malaria, compared with uncomplicated cases and healthy controls (54).
	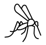	In Brazilian patients with different forms of vivax malaria and controls, a network analysis revealed a protective role of IL-4 and IL-10 in non-infected and asymptomatic patients (55).
**IL-10**	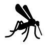	In Beninese children with falciparum cerebral malaria, plasma levels were significantly higher in fatal cases compared with those who survived (58).
	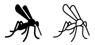	Plasma levels were significantly higher in Rwandan patients with severe malaria, compared with uncomplicated cases and controls (53).
	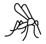	Plasma levels were significantly higher in Colombian patients with vivax severe malaria, compared with uncomplicated cases and healthy controls (54).
	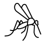	IL-10 production was only observed in Brazilian patients with vivax malaria, compared with previously exposed, non-infected subjects and unexposed healthy donors. Infected patients presented a moderate correlation between IL-10 and IL-6 plasma levels (61).
	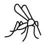	In Brazilian patients with different forms of vivax malaria and controls, a network analysis revealed a protective role of IL-10 and IL-4 in non-infected and asymptomatic vivax patients (55).
	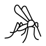	In Pakistani patients with vivax malaria, plasma levels were significantly higher in uncomplicated cases than healthy controls, and higher again in complicated cases compared with uncomplicated ones. IL-10, TNF-α, ICAM-1 and VCAM-1 were the best individual predictors of complicated *vivax* malaria (62).
	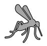	Plasma levels were significantly higher in Malaysian patients with severe knowlesi malaria, compared with uncomplicated cases (63).
**Chemokines**		
**CCL2**	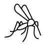	Plasma levels were significantly higher in Colombian patients with vivax severe malaria, compared with uncomplicated cases and healthy controls (54).
	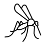	Plasma levels were significantly increased in Brazilian patients with vivax malaria, compared with previously exposed, uninfected subjects. Infected patients showed a strong correlation between CCL2 and IL-6 plasma levels (61).

**CCL4**	Plasma levels were significantly higher in Brazilian patients with acute falciparum and vivax malaria, compared with healthy controls. During the convalescent phase, levels were higher in falciparum malaria patients as compared to vivax cases (71).
**CCL5**	Serum levels were significantly lower in Indian patients with falciparum severe malaria, compared with uncomplicated cases and healthy controls (72).
	Plasma levels were significantly lower in Brazilian patients with vivax malaria, compared with previously exposed, non-infected subjects and unexposed healthy donors (61).
	In Brazilian patients with different forms of vivax malaria and controls, a network analysis revealed that CCL5, IFN-γ, and TNF-α were crucial in the profile of mild malaria cases (55).
**CCL18**	To the best of our knowledge, no studies have reported significant group differences in the context of human malaria.

Other immune markers

Name	Description	Clinical significance
**OPN**	Extracellular matrix bone protein. It can act as a cytokine, enhancing production of IFN-γ and IL-12 and reducing production of IL-10.	Plasma concentrations in Ugandan infants with falciparum malaria were inversely correlated with falciparum-specific atypical memory B cells, suggesting that OPN could have a role in the acquisition of natural immunity against malaria (73).
		Plasma levels were significantly higher in Singapore patients with vascular cognitive impairment, compared with cognitively normal controls (74).
**RAGE**	Cell surface pattern recognition receptor. Triggers a pro-inflammatory response.	To the best of our knowledge, no studies have reported significant group differences in the context of human malaria.

CCL2: chemokine (C-C motif) ligand 2; **CCL4**: chemokine (C-C motif) ligands 4; **CCL5**: chemokine (C-C motif) ligand 5; **CCL18**: chemokine (C-C motif) ligand 18; **CNS**: central nervous system; **CRP**: C-reactive protein; **CSF**: cerebrospinal fluid; **GM-CSF**: granulocyte-macrophage colony-stimulating factor; **IFN-γ**: interferon gamma; **IL-1α**: interleukin 1 alpha; **IL-1β***: interleukin 1 beta; **IL-1RA**: interleukin 1RA; **IL-2**: interleukin 2; **IL-4****: interleukin 4; **IL-6***: interleukin 6; **IL-8**: interleukin 8; **IL-10**: interleukin 10; **IL-17A**: interleukin 17; **ILC**: innate lymphoid cells; **MIF**: migration inhibitory factor; **MPO**: myeloperoxidase; **NK**: natural killer; **OPN**: osteopontin; **RAGE**: receptor for advanced glycation end-products; **TNF-α**: tumour Necrosis Factor alpha; **VSMC**: vascular smooth muscle cells.

*May have anti-inflammatory functions. **May have pro-inflammatory functions.

Legend: Clinical findings related to…

Plasmodium falciparum malaria infection; *Plasmodium vivax* malaria infection; *Plasmodium knowlesi* malaria infection; Neurological conditions.

12 Supplementary Table 3

Vascular biomarkers		
**Name**	**Description**	**Clinical significance**
**Ang-1**	Vascular growth factor. Regulates angiogenesis, endothelial cell survival, and proliferation. Mediates blood vessel maturation and stability.	A decline in Ang-1 plasma levels was associated with increasing falciparum and vivax malaria severity and widespread endothelial activation across African and Asian patient studies, irrespective of age (75–79).
**Ang-2**	Vascular growth factor, biomarker of endothelial activation. Modulates Ang-1 signalling, and in the absence of angiogenic inducers such as VEGF, promotes vascular regression. In concert with VEGF, triggers a permissive angiogenic signal. Involved in lymphangiogenesis.	An increase of Ang-2 plasma levels was associated with increasing falciparum and vivax malaria severity and widespread endothelial activation across African and Asian patient studies, irrespective of age (75,77,79).
		Plasma levels were significantly higher in Malaysian patients with severe knowlesi malaria, compared with uncomplicated cases (63).
		Robust predictor of mortality in falciparum cerebral malaria, identified as a risk factor for blood-brain barrier dysfunction, neuroinflammation, and long-term cognitive injury in African children (78,80,81).
$\frac{\mathbf{Ang2}}{\mathbf{Ang1}}$	Ratio between Ang-2 and Ang-1 plasma levels.	Plasma ratio was higher in patients with falciparum and vivax severe malaria compared with uncomplicated malaria and healthy controls, across African, Asian, and Latin American patient studies and irrespective of age. Fatal cerebral malaria cases showed the highest ratio (75,76,79,82–84).
**BMP-9**	Growth factor, member of the TGF-β superfamily. Regulates angiogenesis by inhibiting VEGF-induced endothelial cell migration and proliferation.	To our knowledge, no studies have reported significant group differences in the context of human malaria.
**ICAM-1**	Intercellular adhesion molecule constitutively expressed on the vascular endothelium. Upon IL-1 and TNF-α stimulation, expression levels increase, so that leukocytes can bind to it and transmigrate into tissues. Increased plasma levels are suggestive of endothelial activation.	Plasma levels were significantly higher in Ugandan children with falciparum severe malaria, compared with healthy controls (78).
		Plasma levels were higher in Malawian children with falciparum cerebral malaria and retinopathy, compared with their retinopathy-negative counterparts (85).
		In Ugandan children with falciparum malaria, plasma levels were elevated in severe malarial anaemia fatalities compared to survivors (86).
		In Ghanian children with falciparum cerebral malaria, plasma levels were higher compared with uncomplicated malaria cases (87).

		In Pakistani patients with vivax malaria, plasma levels were significantly higher in uncomplicated cases than healthy controls, and higher again in complicated cases compared with uncomplicated ones. ICAM-1, VCAM-1, TNF-α and IL-10 were the best individual predictors of complicated *vivax* malaria (62).
		Plasma levels were significantly higher in Malaysian patients with severe knowlesi malaria, compared with uncomplicated cases (63).
**PDGF-AA**	Angiogenic promoter. Plays an important role in wound healing, and it is essential during embryonic development.	Increased plasma levels predicted abnormal cerebral blood flow and stroke in children with sickle cell disease who presented with cerebrovascular disease (88).
**PDGF-BB**	Angiogenic promoter. Participates in wound healing, blood vessel development, and proliferation and recruitment of pericytes and vascular smooth muscle cells in the central nervous system.	In Ugandan children with falciparum cerebral malaria, plasma levels were significantly higher in retinopathy-negative patients, compared to their retinopathy-positive peers (46).
**Serpin E1**	Serine protease inhibitor. Plays a role in the controlled degradation of blood clots.	To our knowledge, no studies have reported significant group differences in the context of human malaria.
**VCAM-1**	Surface glycoprotein expressed on the vascular endothelium. Participates in immune surveillance and inflammation by regulating leukocyte adhesion to the endothelium and transendothelial migration.	Plasma levels were significantly higher in Ugandan children with falciparum severe malaria, compared with healthy controls (78).
		In Pakistani patients with vivax malaria, plasma levels were significantly higher in uncomplicated cases than healthy controls, and higher again in complicated cases compared with uncomplicated ones. VCAM-1, ICAM-1, TNF-α and IL-10 were the best individual predictors of complicated vivax malaria (62).
**VEGF**	Vascular growth factor. Promotes angiogenesis, vasculogenesis, and endothelial cell growth. Induces endothelial cell proliferation, cell migration, and permeabilization of blood vessels.	Plasma levels were significantly higher in Indonesian patients with falciparum malaria compared with healthy controls (89).
		Serum levels were significantly lower in Indian patients with vivax severe malaria compared with uncomplicated malaria and healthy controls (79).
**vWF-A2**	Glycoprotein involved in haemostasis, biomarker of endothelial activation. Promotes adhesion of platelets to sites of vascular injury and acts as a chaperone for certain coagulation factors.	Plasma levels were significantly higher in Ugandan children with falciparum severe malaria, compared with healthy controls (78); increased plasma levels were associated with mortality (90).
		Plasma levels were significantly higher in Malawian children with cerebral malaria than in children with uncomplicated malaria, showing similar values in patients with and without retinopathy (91).

	Plasma levels were significantly increased in Malaysian patients with severe and uncomplicated vivax malaria, compared with controls, and correlated with parasitaemia in these patients (92).

Ang-1: angiopoietin-1; **Ang-2**: angiopoietin-2; **Ang-2/Ang-1**: ratio between Ang-2 and Ang-1; **BMP-9**: bone morphogenetic protein 9; **ICAM-1**: intercellular adhesion molecule 1; **PDGF-AA**: platelet-derived growth factor AA; **PDGF-BB**: platelet-derived growth factor BB; **VCAM-1**: vascular cell adhesion molecule; **Serpin E1**: Serine Proteinase Inhibitor-clade E1; **PEDF**: pigment epithelium derived factor; **VEGF**: vascular endothelial growth factor; **vWF-A2**: von Willebrand Factor (A2 domain).

Legend: Clinical findings related to…

Plasmodium falciparum malaria infection; *Plasmodium vivax* malaria infection; *Plasmodium knowlesi* malaria infection;

Neurological conditions; Other medical conditions.

13 Supplementary Table 4

List of recombinant antigen targets utilised in the Luminex immunoassay to assess malaria exposure				
**Name**	**Gene ID**	**Species**	**Description**	**Location**
*Pk*AMA1	PKNH_0931500	*P. knowlesi*	Apical membrane antigen 1	Merozoite surface
*Pk*MSP1$_{19}$	PKNH_0728900	*P. knowlesi*	Merozoite surface protein 1, 19kD	Merozoite surface
*Pk*Sera3 Ag2	PKNH_0413400	*P. knowlesi*	Cysteine protease (Serine repeat-like antigen)	Unknown
*Pk*SSP2/TRAP	PKNH_1265400	*P. knowlesi*	Sporozoite surface protein 2, putative, thrombospondin-related anonymous protein (TRAP)	Sporozoite surface
*Pk*1 (*Pk*CSP_F)	PKNH_1325300	*P. knowlesi*	Hypothetical protein	Unknown
*Pk*8	PKNH_0400300	*P. knowlesi*	Plasmodium exported protein, unknown function	Unknown
*Pv*AMA1	PVX_092275	*P. vivax*	Apical membrane antigen 1	Merozoite surface
*Pv*MSP1$_{19}$	PVX_099980	*P. vivax*	Merozoite surface protein ,1, 19kD	Merozoite surface
*Pv*RBP2b	PVX_094255	*P. vivax*	Reticulocyte binding protein 2b fragment	Merozoite micronemes
*Pf*AMA1	PF3D7_1133400	*P. falciparum*	Apical membrane antigen 1	Sporozoite / merozoite surface
*Pf*MSP1$_{19}$	PF3D7_0930300	*P. falciparum*	Merozoite surface protein 1, 19kD	Merozoite surface
Etramp5 Ag1	PF3D7_0532100	*P. falciparum*	Early transcribed membrane protein 5 antigen 1	Infected erythrocyte / parasitophorous vacuole membrane
*Po*MSP1$_{19}$	PocGH01_07037900	*P. ovale*	Merozoite surface protein 1, 19kD	Merozoite surface
*Pm*MSP1$_{19}$	PmUG01_07042000	*P. malariae*	Merozoite surface protein 1, 19kD	Merozoite surface
Tetanus toxoid	--	*Clostridium tetani*	Inactivated tetanus toxin immunisation antigen; internal human control	--
GST	GST26_SCHJA	*Schistosoma japonicum*	Gluthanoid-S-transferase purification tag; GST-tagged protein control	--

14 Supplementary Table 5

Individual biomarker group comparisons (Wilcoxon test)

		**Healthy controls** (N=19) Median pg/mL (IQR)	***Pk*-infected patients** (N=19) Median pg/mL (IQR)	**Crude P value**	**Corrected[ϕ] P value**
	**αSyn**	863.4 (259.9)	2,177.8 (392.4)	**<0.0001**	**<0.0001**
	**APP**	36,082.0 (25,945.0)	17,701 (12,728.0)	**0.0010**	0.05
**Biomarkers of brain alterations or cerebral injury**	**Aβ$_{(1-42)}$**	0.2 (1.2)	1.6 (0.9)	0.08	1.00
	**BDNF**	1,516.2 (1,454.1)	146.2 (115.8)	**<0.0001**	**<0.0001**
	**CaBD**	1,807.0 (179.0)	1,151.1 (221.0)	**<0.0001**	**<0.0001**
	**CNTN1**	18,191.0 (4,890.0)	6,179.7 (2,615.6)	**<0.0001**	**<0.0001**
	**ENO2/NSE***	54,324.0 (21,601.0)	1,094,366.0 (1,382,298.0)	**0.0010**	0.05
	**Fetuin A**	176,390,803.0 (38,746,703.0)	154,460,943.0 (47,209,645.0)	**0.0462**	1.00
	**GFAP***	78.1 (45.8)	29.9 (25.3)	**<0.0001**	**0.0013**
	**KLK6**	2,319.5 (915.0)	1,072.4 (788.7)	**0.0003**	**0.0126**
	**Lipocalin 2**	547,608.0 (149,670.0)	619,695.0 (94,135.0)	0.05	1.00
	**NCAM-1**	107,813.0 (38,204.0)	70,203.0 (8,851.0)	**<0.0001**	**<0.0001**
	**NfL**	8.1 (4.4)	6.3 (5.8)	0.21	1.00
	**NGF-β***	0.9 (1.2)	1.7 (1.5)	**0.0203**	1.00
	**NRGN***	22.4 (11.1)	241.4 (763.1)	**<0.0001**	**0.0022**
	**Park7**	71,436.0 (61,372.0)	161,810.0 (146,171.0)	**<0.0001**	**0.0006**
	**S100B**	27.3 (0.0)	1,282.2 (1,777.2)	**<0.0001**	**<0.0001**
	**Tau total**	0.6 (0.6)	3. 7 (5.0)	**<0.0001**	**0.0007**
	**TDP-43***	5,344.3 (5,806.0)	67,143.0 (90,407.0)	**<0.0001**	**0.0050**
	**UCHL-1**	49.3 (45. 4)	336.6 (184.1)	**<0.0001**	**<0.0001**
	**YKL40**	29,591.0 (24,732.0)	58,674.0 (33,187.0)	**0.0020**	0.10

[ϕ]Bonferroni correction for multiple comparisons. *Values out of range: ENO2/ NSE (n=3 in the *Pk*-infected group); GFAP (n=1 in the *Pk*-infected group); NGF-β (n=2 in the control group); NRGN (n=1 in the *Pk*-infected group); TDP-43 (n=2 in the control group).

		Healthy controls (N=19) Median pg/mL (IQR)	*Pk*-infected patients (N=19) Median pg/mL (IQR)	Crude P value	Corrected[ϕ] P value
Biomarkers of infection and immune activation	Pro-inflammatory cytokines	**CRP**	668,496.0 (308,499.0)	477,838.0 (235,914.0)	**0.0005**	**0.0280**
		**GM-CSF**	23.8 (10.0)	21.52 (8.7)	0.24	1.00
		**IFNγ**	101.4 (11.8)	104.8 (46.3)	0.73	1.00
		**IL-1α**	49.0 (46.4)	46.0 (21.2)	0.29	1.00
		**IL-1β**	24.7 (7.2)	40.0 (17.9)	**0.0027**	0.14
		**IL-2**	34.8 (28.7)	33.1 (62.5)	0.53	1.00
		**IL-6***	11.5 (2.6)	21.7 (19.2)	**0.0013**	0.06
		**IL-8**	319.7 (599.0)	254.6 (2,353.2)	0.82	1.00
		**IL-17A**	29.2 (6.1)	22.2 (8.3)	**0.0081**	0.41
		**MIF**	384.6 (315.8)	630.7 (321.2)	**0.0022**	0.11
		**MPO**	1,571,073.0 (869,998.0)	3,176,104.0 (919,812.0)	**0.0006**	**0.0314**
		**TNF-α**	16.2 (5.7)	13.7 (2.3)	**0.0393**	1.00
	Anti-	**IL-1RA**	6,736.0 (2,506.0)	35,639.0 (21,569.0)	**<0.0001**	**<0.0001**
		**IL-4**	165.1 (12.2)	159.8 (35.1)	0.44	1.00
		**IL-10**	8.0 (1.3)	48.6 (116.0)	**<0.0001**	**<0.0001**
	Chemokines	**CCL2**	380.8 (326.0)	160.9 (219.4)	**0.0075**	0.38
		**CCL4**	781.8 (250.0)	526.3 (47.7)	**<0.0001**	**0.0002**
		**CCL5**	158,730.0 (96,050.0)	17,650.0 (27,045.0)	**<0.0001**	**<0.0001**
		**CCL18**	76,800.0 (37,565.0)	76,703.9 (73,970.5)	0.89	1.00
	Other	**OPN**	12,981.0 (11,051.0)	5,057.0 (3,291.0)	**0.0033**	0.17
		**RAGE**	7,206.0 (2,599.0)	4,797.0 (1,840.0)	**<0.0001**	**0.0025**

[ϕ]Bonferroni correction for multiple comparisons. *Values out of range: IL-6 (n=1 in the *Pk*-infected group).

		Healthy controls (N=19) Median pg/mL (IQR)	*Pk*-infected patients (N=19) Median pg/mL (IQR)	Crude P value	Corrected[ϕ] P value
Vascular biomarkers	**Ang-1**	51,807.0 (23,226.0)	1,547.8 (1,633.8)	**<0.0001**	**<0.0001**
	**Ang-2**	3,187.0 (1,277.0)	2,561.7 (1,365.2)	**0.0471**	1.00
	**Ang-2/Ang-1 ratio**	0.1 (0.1)	1. 5 (0.6)	**<0.0001**	**<0.0001**
	**BMP-9**	205.9 (193.2)	3.6 (2.3)	**<0.0001**	**<0.0001**
	**ICAM-1**	705,782.0 (710,688.0)	832,537.0 (324,397.0)	0.98	1.00
	**PDGF AA**	3,864.0 (689.0)	552.6 (346.2)	**<0.0001**	**<0.0001**
	**PDGF BB***	18,495.0 (7,646.0)	1,385.7 (2,055.8)	**<0.0001**	**<0.0001**
	**Serpin E1**	932,450.0 (468,184.0)	183,488.0 (103,411.0)	**<0.0001**	**<0.0001**
	**VCAM-1**	698,022.0 (419,633.0)	1,673,430.0 (2,154,222.0)	**<0.0001**	**0.0001**
	**VEGF**	147.3 (81.2)	178.9 (118.7)	0.26	1.00
	**vWF**	6,716.8 (8,770.7)	6,173.0 (3,737.2)	0.20	1.00

[ϕ]Bonferroni correction for multiple comparisons. *Values out of range: PDGF BB (n=1 in the *Pk*-infected group).

Biomarkers of brain alterations or cerebral injury:

αSyn: alpha-Synuclein; **APP**: amyloid-beta precursor protein; **A$\beta_{1\text{-}42}$**: amyloid beta (1-42); **BDNF**: brain-derived neurotrophic factor; **CaBD**: Calbindin D; **CNTN1**: contactin-1; **CSF**: cerebrospinal fluid; **ENO2** / **NSE**: Enolase 2 / Neuron-specific Enolase; **GFAP**: glial fibrillary acidic protein; **KLK6**: kallikrein 6 / neurosin; **NCAM-1**: neural cell adhesion molecule; **Lipocalin-2**: neutrophil gelatinase-associated lipocalin; **NGF-β**: nerve growth factor beta; **NfL**: neurofilament light chain; **NRGN**: neurogranin; **Park7**: Parkinsonism-associated deglycase; **S100B**: S100 calcium-binding protein β; **TDP-43**: TAR DNA-binding protein 43; **Tau**: total Tau protein; **UCH-L1**: ubiquitin carboxy-terminal hydrolase L1; **YKL40**: Chitinase-3-like protein 1.

Biomarkers of infection and immune activation:

CCL2: chemokine (C-C motif) ligand 2; **CCL4**: chemokine (C-C motif) ligands 4; **CCL5**: chemokine (C-C motif) ligand 5; **CCL18**: chemokine (C-C motif) ligand 18; **CRP**: C-reactive protein; **GM-CSF**: granulocyte-macrophage colony-stimulating factor; **IFN-γ**: interferon gamma; **IL-1α**: interleukin 1 alpha; **IL-1β**: interleukin 1 beta; **IL-1RA**: interleukin 1RA; **IL-2**: interleukin 2; **IL-4**: interleukin 4; **IL-6**: interleukin 6; **IL-8**: interleukin 8; **IL-10**: interleukin 10; **IL-17A**: interleukin 17; **MIF**: migration inhibitory factor; **MPO**: myeloperoxidase; **OPN**: osteopontin; **RAGE**: receptor for advanced glycation end-products; **TNF-α**: tumour Necrosis Factor alpha.

Vascular biomarkers:

Ang-1: angiopoietin-1; **Ang-2**: angiopoietin-2; **Ang-2/Ang-1**: ratio between Ang-2 and Ang-1; **BMP-9**: bone morphogenetic protein 9; **ICAM-1**: intercellular adhesion molecule 1; **PDGF-AA**: platelet-derived growth factor AA; **PDGF-BB**: platelet-derived growth factor BB; **Serpin E1**: Serine Proteinase Inhibitor-clade E1; **VCAM-1**: vascular cell adhesion molecule; **VEGF**: vascular endothelial growth factor; **vWF-A2**: von Willebrand Factor (A2 domain). ***Pk***: *Plasmodium knowlesi*; **IQR**: interquartile range.

15 Supplementary Table 6

Clinical data extracted from the parent study: *Plasmodium knowlesi*-infected patients

State	Sex	Age	Parasitaemia	Information (translated from Malay)	Lactate (mmol/L)	
Johor	Male	31	0.0082	A soldier	Insufficient sample	
	Male		0.0944	Patient works as a farmer, transporting palm oil every day except Sundays. Working hours are from 6:00 am to 6:30 pm. Movement only around the farm. Usually only comes to town at the beginning of the month to buy necessities and withdraw their monthly salary.<br>Two days ago, symptoms and signs started to appear: night-time shivers, body aches, and fever. Patient stayed on the farm and worked as usual. After two days, the fever wasn't going down so the patient decided to go to the hospital. Blood film for malaria parasite was done and the patient was confirmed to have malaria (*Plasmodium knowlesi*). Treatment was provided immediately.	Insufficient sample	
	Male	31	0.0208	Patient came back from another state, where he believes to have acquired the infection. Patient was on duty at for a month, plus on a 7-day operation. Visited his father in another state and was involved in umrah (pilgrimage) for half a month.	**10.42**	Above normal range
Pahang	Male	33	0.0586	Patient works as a wildlife ranger in a nature reserve. Diagnosed with *Plasmodium knowlesi* **uncomplicated malaria**, 2930/0 μL/ blood, onset symptoms were chills and rigors.	Insufficient sample	
	Male	32	0.5184	Patient diagnosed with knowlesi **severe malaria** (zoonotic) with onset symptoms of chills, vomiting and headache, 25,920/0 μl/ blood.<br>Patient works as rubber tapper. Reports entering the forest area and spending the night hunting without using repellant or personal protection equipment.	Insufficient sample	
	Male	54	1.9520	Patient reports a few days ago with symptoms of chills and rigor, plus on and off fever, myalgia, arthralgia, and fever. Diagnosed with *P. knowlesi* **severe malaria**, 97,600/0 μl/ blood.<br>Occupation: Miner. Risk of Infection: Gardening on a small scale around the house area until late at dusk (between 5:30 and 7:00 pm), The house is on the edge of the forest, with presence of primates around the residence.	Insufficient sample	
	Male	32	0.5405	Patient reports symptoms a few days ago. Diagnosed with *P knowlesi* **severe malaria**, 27,027/0 μl/ blood.<br>Occupation: Farm worker. Risk of infection: Living and working in a risk area.	Insufficient sample	
	Male	35	0.0240	Onset symptoms were headache, chills and rigors. Diagnosed with *Plasmodium knowlesi* **uncomplicated malaria**, 1200/0 μl/blood).<br>Risk activities are hunting and fishing.	Insufficient sample	

Perak	Male	28	0.2208	(*No registered clinical history*)	**11.31**	Above normal range
	Male	31	0.0827	(*No registered clinical history*)	**9.50**	Above normal range
Selangor	Male	79	0.6784	Patient works as gardener and has no history of going into jungle or forested areas. There have been macaques sighting at the school he works at.	Insufficient sample	
	Male	57	4.5440	Work as a security guard at a school located near to a forest. Just came out from the forest and diagnosed with knowlesi malaria. Patient is also positive for dengue IgG.	Insufficient sample	
	Male	26	0.0664	History: Went to the jungle a day before. Symptoms of fever, headache, myalgia, and arthralgia.	Insufficient sample	
	Male	30	0.4453	Indigenous patient	Insufficient sample	
	Male	46	8.7771	(*No registered clinical history*)	Insufficient sample	
	Male	63	0.3040	(*No registered clinical history*)	Insufficient sample	
	Male	24	0.4459	Visited the river area	Insufficient sample	
	Male	32	0.8808	Fever, backpain, lethargy, headache	1.93	Within normal range
Trengganu	Male	29	0.2504	Jungle trekking	Insufficient sample	

Age is expressed in years

16 References

1. Herman LS, Fornace K, Phelan J, Grigg MJ, Anstey NM, William T, *et al*. Identification and validation of a novel panel of *Plasmodium knowlesi* biomarkers of serological exposure. *PLoS Negl Trop Dis* (2018) 12(6):e0006457.

2. Fornace KM, Herman LS, Abidin TR, Chua TH, Daim S, Lorenzo PJ, *et al*. Exposure and infection to *Plasmodium knowlesi* in case study communities in Northern Sabah, Malaysia and Palawan, The Philippines. *PLoS Negl Trop Dis* (2018) 12(6):e0006432.

3. Fornace KM, Brock PM, Abidin TR, Grignard L, Herman LS, Chua TH, *et al*. Environmental risk factors and exposure to the zoonotic malaria parasite *Plasmodium knowlesi* across northern Sabah, Malaysia: a population-based cross-sectional survey. *Lancet Planet Health* (2019) 3(4):e179–86.

4. Longley RJ, White MT, Takashima E, Brewster J, Morita M, Harbers M, *et al*. Development and validation of serological markers for detecting recent *Plasmodium vivax* infection. *Nature Medicine* (2020) 26(5):741–9.

5. Drakeley CJ, Corran PH, Coleman PG, Tongren JE, McDonald SLR, Carneiro I, *et al*. Estimating medium- and long-term trends in malaria transmission by using serological markers of malaria exposure. *Proc Natl Acad Sci USA* (2005) 102(14):5108–13.

6. Rogier E, Nace D, Dimbu PR, Wakeman B, Pohl J, Beeson JG, *et al*. Framework for Characterizing Longitudinal Antibody Response in Children After *Plasmodium falciparum* Infection. *Front Immunol* (2021) 12:617951.

7. Tetteh KKA, Wu L, Hall T, Ssewanyana I, Oulton T, Patterson C, *et al*. Optimisation and standardisation of a multiplex immunoassay of diverse *Plasmodium falciparum* antigens to assess changes in malaria transmission using sero-epidemiology. *Wellcome Open Res* (2020) 4:26.

8. Fernandez-Camacho B, Peña-Calero B, Guillermo-Roman M, Ruiz-Cabrejos J, Barboza JL, Bartolini-Arana L, *et al*. Malaria seroepidemiology in very low transmission settings in the Peruvian Amazon. *Scientific Reports* (2024) 14(1):1–14.

9. Pinkevych M, Petravic J, Chelimo K, Kazura JW, Moormann AM, Davenport MP. The Dynamics of Naturally Acquired Immunity to *Plasmodium falciparum* Infection. *PLoS Comput Biol* (2012) 8(10):e1002729.

10. Zubelzu M, Morera-Herreras T, Irastorza G, Gómez-Esteban JC, Murueta-Goyena A. Plasma and serum alpha-synuclein as a biomarker in Parkinson's disease: A meta-analysis. *Parkinsonism Relat Disord* (2022) 99:107–15.

11. Chiu PY, Yang FC, Chiu MJ, Lin WC, Lu CH, Yang SY. Relevance of plasma biomarkers to pathologies in Alzheimer's disease, Parkinson's disease and frontotemporal dementia. *Scientific Reports* (2022) 12(1):1–9.

12. Nakamura A, Kaneko N, Villemagne VL, Kato T, Doecke J, Doré V, *et al*. High performance plasma amyloid-β biomarkers for Alzheimer's disease. *Nature* (2018) 249–54.

13. Cheng L, Li W, Chen Y, Lin Y, Wang B, Guo Q, *et al*. Plasma Aβ as a biomarker for predicting Aβ-PET status in Alzheimer's disease: a systematic review with meta-analysis. *J Neurol Neurosurg Psychiatry* (2022) 93(5):513–20.

14. McDonald CR, Conroy AL, Hawkes M, Elphinstone RE, Gamble JL, Hayford K, *et al*. Brain-derived neurotrophic factor is associated with disease severity and clinical outcome in Ugandan children admitted to hospital with severe malaria. *Pediatric Infectious Disease Journal* (2017) 36(2):146–50.

15. Craig-Schapiro R, Kuhn M, Xiong C, Pickering EH, Liu J, Misko TP, et al. Multiplexed Immunoassay Panel Identifies Novel CSF Biomarkers for Alzheimer's Disease Diagnosis and Prognosis. *PLoS One* (2011) 6(4):e18850.

16. Abdi IY, Bartl M, Dakna M, Abdesselem H, Majbour N, Trenkwalder C, *et al*. Cross-sectional proteomic expression in Parkinson's disease-related proteins in drug-naïve patients vs healthy controls with longitudinal clinical follow-up. *Neurobiol Dis* (2023) 177:105997.

17. Thelin EP, Zeiler FA, Ercole A, Mondello S, Büki A, Bellander BM, *et al*. Serial sampling of serum protein biomarkers for monitoring human traumatic brain injury dynamics: A systematic review. *Front Neurol* (2017) 8(7):277421.

18. Rodríguez-Rodríguez A, Egea-Guerrero JJ, Gordillo-Escobar E, Enamorado-Enamorado J, Hernández-García C, Ruiz de Azúa-López Z, *et al*. S100B and Neuron-Specific Enolase as mortality predictors in patients with severe traumatic brain injury. *Neurol Res* (2016) 38(2):130–7.

19. Böhmer AE, Oses JP, Schmidt AP, Perón CS, Krebs CL, Oppitz PP, *et al*. Neuron-specific enolase, S100B, and glial fibrillary acidic protein levels as outcome predictors in patients with severe traumatic brain injury. *Neurosurgery* (2011) 68(6):1624–30.

20. Chen Y, Chan CK, Kerishnan JP, Lau YL, Wong YL, Gopinath SCB. Identification of circulating biomarkers in sera of *Plasmodium knowlesi*-infected malaria patients - comparison against *Plasmodium vivax* infection. *BMC Infect Dis* (2015) 15(1):1–10.

21. Bazarian JJ, Biberthaler P, Welch RD, Lewis LM, Barzo P, Bogner-Flatz V, *et al*. Serum GFAP and UCH-L1 for prediction of absence of intracranial injuries on head CT (ALERT-TBI): a multicentre observational study. L*ancet Neurol* (2018) 17(9):782–9.

22. Eide PK, Lashkarivand A, Pripp A, Valnes LM, Hovd MH, Ringstad G, *et al*. Plasma neurodegeneration biomarker concentrations associate with glymphatic and meningeal lymphatic measures in neurological disorders. *Nature Communications* (2023) 14(1):1–14.

23. Shen XN, Huang SY, Cui M, Zhao QH, Guo Y, Huang YY, *et al*. Plasma Glial Fibrillary Acidic Protein in the Alzheimer Disease Continuum: Relationship to Other Biomarkers, Differential Diagnosis, and Prediction of Clinical Progression. *Clin Chem* (2023) 69(4):411–21.

24. Chouliaras L, Thomas A, Malpetti M, Donaghy P, Kane J, Mak E, *et al*. Differential levels of plasma biomarkers of neurodegeneration in Lewy body dementia, Alzheimer's disease, frontotemporal dementia and progressive supranuclear palsy. *J Neurol Neurosurg Psychiatry* (2022) 93(6):651–8.

25. Patra K, Soosaipillai A, Sando SB, Lauridsen C, Berge G, Møller I, *et al*. Assessment of kallikrein 6 as a cross-sectional and longitudinal biomarker for Alzheimer's disease. *Alzheimers Res Ther* (2018) 10(1):1–11.

26. Mahamar A, Gonzales Hurtado PA, Morrison R, Boone R, Attaher O, Diarra BS, *et al*. Plasma biomarkers of hemoglobin loss in *Plasmodium falciparum*–infected children identified by quantitative proteomics. *Blood* (2022) 139(15):2361–76.

27. Sahu PK, Hoffmann A, Majhi M, Pattnaik R, Patterson C, Mahanta KC, *et al*. Brain Magnetic Resonance Imaging Reveals Different Courses of Disease in Pediatric and Adult Cerebral Malaria. *Clinical Infectious Diseases* (2021) 73(7):e2387–96.

28. Li X, Wang X, Guo L, Wu K, Wang L, Rao L, *et al*. Association between lipocalin-2 and mild cognitive impairment or dementia: A systematic review and meta-analysis of population-based evidence. *Ageing Res Rev* (2023) 89:101984.

29. Shi Y, Luan D, Song R, Zhang Z. Value of peripheral neurotrophin levels for the diagnosis of depression and response to treatment: A systematic review and meta-analysis. *European Neuropsychopharmacology* (2020) 41:40–51.

30. Qin XY, Wu HT, Cao C, Loh YP, Cheng Y. A meta-analysis of peripheral blood nerve growth factor levels in patients with schizophrenia. *Molecular Psychiatry* (2017) 22(9):1306–12.

31. Datta D, Gopinadhan A, Soto A, Bangirana P, Opoka RO, Conroy AL, *et al*. Blood biomarkers of neuronal injury in paediatric cerebral malaria and severe malarial anaemia. *Brain Commun* (2023) 5(6).

32. Balanza N, Francis CK, Crowley VM, Weckman AM, Zhong K, Baro B, *et al*. Neurofilament Light Chain as a Biomarker of Neuronal Damage in Children With Malaria. *J Infect Dis* (2024) 229(1):183–8.

33. Gao W, Zhang Z, Lv X, Wu Q, Yan J, Mao G, *et al*. Neurofilament light chain level in traumatic brain injury: A system review and meta-analysis. *Medicine* (2020) 99(38):e22363.

34. Zhao Y, Xin Y, Meng S, He Z, Hu W. Neurofilament light chain protein in neurodegenerative dementia: A systematic review and network meta-analysis. *Neurosci Biobehav Rev* (2019) 102:123–38.

35. Dietrick B, Molloy E, Massaro AN, Strickland T, Zhu J, Slevin M, et al. Plasma and Cerebrospinal Fluid Candidate Biomarkers of Neonatal Encephalopathy Severity and Neurodevelopmental Outcomes. *Journal of Pediatrics* (2020) 226:71-79.e5.

36. Yang J, Korley FK, Dai M, Everett AD. Serum neurogranin measurement as a biomarker of acute traumatic brain injury. *Clin Biochem* (2015) 48(13–14):843–8.

37. Çevik S, Özgenç MM, Güneyk A, Evran Ş, Akkaya E, Çalış F, *et al*. NRGN, S100B and GFAP levels are significantly increased in patients with structural lesions resulting from mild traumatic brain injuries. *Clin Neurol Neurosurg* (2019) 183:105380.

38. Katunina EA, Blokhin V, Nodel MR, Pavlova EN, Kalinkin AL, Kucheryanu VG, *et al*. Searching for Biomarkers in the Blood of Patients at Risk of Developing Parkinson's Disease at the Prodromal Stage. *Int J Mol Sci* (2023) 24(3):1842.

39. Rogan A, O'Sullivan MB, Holley A, McQuade D, Larsen P. Can serum biomarkers be used to rule out significant intracranial pathology in emergency department patients with mild traumatic brain injury? A Systemic Review & Meta-Analysis. *Injury* (2022) 53(2):259–71.

40. Michetti F, D'Ambrosi N, Toesca A, Puglisi MA, Serrano A, Marchese E, *et al*. The S100B story: from biomarker to active factor in neural injury. *J Neurochem* (2019) 148(2):168–87.

41. Wang F, Zou ZR, Yuan D, Gong Y, Zhang L, Chen X, *et al*. Correlation between serum S100β protein levels and cognitive dysfunction in patients with cerebral small vessel disease: A case-control study. *Biosci Rep* (2017) 37(2).

42. Mohanty S, Sahu PK, Pattnaik R, Majhi M, Maharana S, Bage J, *et al*. Evidence of Brain Alterations in Noncerebral Falciparum Malaria. *Clinical Infectious Diseases* (2022) 75(1):11–8.

43. Katisko K, Huber N, Kokkola T, Hartikainen P, Krüger J, Heikkinen AL, *et al*. Serum total TDP-43 levels are decreased in frontotemporal dementia patients with C9orf72 repeat expansion or concomitant motoneuron disease phenotype. *Alzheimers Res Ther* (2022) 14(1):1–10.

44. Ren Y, Li S, Chen S, Sun X, Yang F, Wang H, *et al*. TDP-43 and Phosphorylated TDP-43 Levels in Paired Plasma and CSF Samples in Amyotrophic Lateral Sclerosis. *Front Neurol* (2021) 12:663637.

45. Datta D, Bangirana P, Opoka RO, Conroy AL, Co K, Bond C, *et al*. Association of Plasma Tau With Mortality and Long-term Neurocognitive Impairment in Survivors of Pediatric Cerebral Malaria and Severe Malarial Anemia. *JAMA Netw Open* (2021) 4(12):e2138515–e2138515.

46. Villaverde C, Namazzi R, Shabani E, Park GS, Datta D, Hanisch B, *et al*. Retinopathy-Positive Cerebral Malaria Is Associated With Greater Inflammation, Blood-Brain Barrier Breakdown, and Neuronal Damage Than Retinopathy-Negative Cerebral Malaria. *J Pediatric Infect Dis Soc* (2020) 9(5):580–6.

47. Datta D, Conroy AL, Castelluccio PF, Ssenkusu JM, Park GS, Opoka RO, *et al*. Elevated Cerebrospinal Fluid Tau Protein Concentrations on Admission Are Associated With Long-term Neurologic and Cognitive Impairment in Ugandan Children With Cerebral Malaria. *Clinical Infectious Diseases* (2020) 70(6):1161–8.

48. Erdman LK, Petes C, Lu Z, Dhabangi A, Musoke C, Cserti-Gazdewich CM, *et al*. Chitinase 3-like 1 is induced by *Plasmodium falciparum* malaria and predicts outcome of cerebral malaria and severe malarial anaemia in a case-control study of African children. *Malar J* (2014) 13(1):1–11.

49. Conroy AL, Hawkes MT, Elphinstone R, Opoka RO, Namasopo S, Miller C, *et al*. Chitinase-3-like 1 is a biomarker of acute kidney injury and mortality in paediatric severe malaria. *Malar J* (2018) 17(1):1–11.

50. Kho S, Anstey NM, Barber BE, *et al*. Diagnostic performance of a 5-plex malaria immunoassay in regions co-endemic for *Plasmodium falciparum*, *P. vivax*, *P. knowlesi*, *P. malariae* and *P. ovale*. *Scientific Reports* (2022) 12(1):1–9.

51. Peto TJ, Tripura R, Lee SJ, *et al*. Association between Subclinical Malaria Infection and Inflammatory Host Response in a Pre-Elimination Setting. *PLoS One* (2016) 11(7):e0158656.

52. Lima-Junior J da C, Rodrigues-da-Silva RN, Pereira VA, *et al*. Cells and mediators of inflammation (C-reactive protein, nitric oxide, platelets and neutrophils) in the acute and convalescent phases of uncomplicated *Plasmodium vivax* and *Plasmodium falciparum* infection. *Mem Inst Oswaldo Cruz* (2012) 107(8):1035–41.

53. Ndoricyimpaye EL, Van Snick J, Niyoyita J de D, *et al*. Integrated Analysis of Cytokine Profiles in Malaria Patients Discloses Selective Upregulation of TGF-β1, β3, and IL-9 in Mild Clinical Presentation. *Int J Mol Sci* (2022) 23(20):12665.

54. Acero CT, Ramírez-Montoya J, Velasco MC, *et al*. IL-4, IL-10, CCL2 and TGF-β as potential biomarkers for severity in Plasmodium vivax malaria. *PLoS Negl Trop Dis* (2022) 16(9):e0010798.

55. Mendonça VR, Queiroz AT, Lopes FM, *et al*. Networking the host immune response in *Plasmodium vivax* malaria. *Malar J* (2013) 12(1):1–11.

56. Mahittikorn A, Kwankaew P, Rattaprasert P, *et al*. Elevation of serum interleukin-1β levels as a potential indicator for malarial infection and severe malaria: a meta-analysis. *Malar J* (2022) 21(1):1–11.

57. Prakash D, Fesel C, Jain R, *et al*. Clusters of cytokines determine malaria severity in *Plasmodium falciparum*-infected patients from endemic areas of Central India. *J Infect Dis* (2006) 194(2):198–207.

58. Royo J, Vianou B, Accrombessi M, *et al*. Elevated plasma interleukin-8 as a risk factor for mortality in children presenting with cerebral malaria. *Infect Dis Poverty* (2023) 12(1):1–15.

59. Moncunill G, Mayor A, Bardají A, *et al*. Cytokine Profiling in Immigrants with Clinical Malaria after Extended Periods of Interrupted Exposure to *Plasmodium falciparum*. *PLoS One* (2013) 8(8):e73360.

60. Moncunill G, Mayor A, Jiménez A, *et al*. Cytokine and Antibody Responses to *Plasmodium falciparum* in Naïve Individuals during a First Malaria Episode: Effect of Age and Malaria Exposure. *PLoS One* (2013) 8(2):e55756.

61. Hojo-Souza NS, Pereira DB, De Souza FSH, *et al*. On the cytokine/chemokine network during *Plasmodium vivax* malaria: new insights to understand the disease. *Malar J* (2017) 16(1):1–10.

62. Raza A, Ghanchi NK, Zubairi ABS, *et al*. Tumor Necrosis Factor -α, Interleukin-10, Intercellular and Vascular Adhesion Molecules Are Possible Biomarkers of Disease Severity in Complicated *Plasmodium vivax* Isolates from Pakistan. *PLoS One* (2013) 8(12):e81363.

63. Barber BE, Grigg MJ, William T, *et al*. Effects of Aging on Parasite Biomass, Inflammation, Endothelial Activation, Microvascular Dysfunction and Disease Severity in *Plasmodium knowlesi* and *Plasmodium falciparum* Malaria. *J Infect Dis* (2017) 215(12):1908–17.

64. Obeng-Aboagye E, Frimpong A, Amponsah JA, *et al*. Inflammatory cytokines as potential biomarkers for early diagnosis of severe malaria in children in Ghana. *Malar J* (2023) 22(1):1–8.

65. Herbert F, Tchitchek N, Bansal D, *et al*. Evidence of IL-17, IP-10, and IL-10 involvement in multiple-organ dysfunction and IL-17 pathway in acute renal failure associated to *Plasmodium falciparum* malaria. *J Transl Med* (2015) 13(1):1–11.

66. Han C, Lin Y, Shan G, *et al*. Plasma concentration of malaria parasite-derived macrophage migration inhibitory factor in uncomplicated malaria patients correlates with parasitemia and disease severity. Clin Vaccine Immunol (2010) 17(10):1524–32.

67. Ngum NH, Fakeh NB, Lem AE, *et al*. Prevalence of malaria and associated clinical manifestations and myeloperoxidase amongst populations living in different altitudes of Mezam division, North West Region, Cameroon. *Malar J* (2023) 22(1):1–14.

68. Kinra P, Dutta V. Serum TNF alpha levels: a prognostic marker for assessment of severity of malaria. *Trop Biomed*. 2013;30(4):645–53.

69. Mandala WL, Msefula CL, Gondwe EN, *et al*. Monocyte activation and cytokine production in Malawian children presenting with *P. falciparum* malaria. *Parasite Immunol* (2016) 38(5):317–25.

70. Cox-Singh J, Singh B, Daneshvar C, *et al*. Anti-Inflammatory Cytokines Predominate in Acute Human *Plasmodium knowlesi* Infections. *PLoS One* (2011) 6(6):e20541.

71. Rodrigues-da-Silva RN, Lima-Junior J da C, Fonseca e Fonseca B de P, *et al*. Alterations in cytokines and haematological parameters during the acute and convalescent phases of *Plasmodium falciparum* and *Plasmodium vivax* infections. *Mem Inst Oswaldo Cruz* (2014) 109(2):154–62.

72. Bujarbaruah D, Kalita MP, Baruah V, *et al.* RANTES levels as a determinant of falciparum malaria severity or recovery. *Parasite Immunol* (2017) 39(9):e12452.

73. Mortazavi SE, Lugaajju A, Kaddumukasa M, *et al*. Osteopontin and malaria: no direct effect on parasite growth, but correlation with *P. falciparum*-specific B cells and BAFF in a malaria endemic area. *BMC Microbiol* (2021) 21(1):1–13.

74. Chai YL, Chong JR, Raquib AR, *et al*. Plasma osteopontin as a biomarker of Alzheimer's disease and vascular cognitive impairment. *Sci Rep* (2021) 11(1):1–11.

75. De Jong GM, Slager JJ, Verbon A, *et al*. Systematic review of the role of angiopoietin-1 and angiopoietin-2 in *Plasmodium* species infections: biomarkers or therapeutic targets? *Malar J* (2016) 15(1):1–12.

76. Jain V, Lucchi NW, Wilson NO, *et al*. Plasma levels of angiopoietin-1 and -2 predict cerebral malaria outcome in Central India. *Malar J* (2011) 10(1):1–7.

77. Conroy AL, Glover SJ, Hawkes M, *et al*. Angiopoietin-2 levels are associated with retinopathy and predict mortality in Malawian children with cerebral malaria: A retrospective case-control study. *Crit Care Med* (2012) 40(3):952–9.

78. Ouma BJ, Ssenkusu JM, Shabani E, *et al*. Endothelial Activation, Acute Kidney Injury, and Cognitive Impairment in Pediatric Severe Malaria. *Crit Care Med* (2020) 48(9):E734–43.

79. Gowda S, Anghan H, Mishra H, *et al*. Serum Angiopoietin-1 and -2 and VEGF are associated with severe disease in vivax malaria. *J Vector Borne Dis* (2020) 57(4):285–94.

80. Petersen JEV, Mkumbaye SI, Vaaben A V, *et al*. Plasma Ang2 and ADAM17 levels are elevated during clinical malaria; Ang2 level correlates with severity and expression of EPCR-binding PfEMP1. *Scientific Reports* (2016) 6(1):1–9.

81. Ouma BJ, Bangirana P, Ssenkusu JM, et al. Plasma angiopoietin-2 is associated with age-related deficits in cognitive sub-scales in Ugandan children following severe malaria. *Malar J* (2021) 20(1):1–10.

82. Gomes LT, Alves- ER, Rodrigues-Jesus C, *et al*. Angiopoietin-2 and Angiopoietin-2/Angiopoietin-1 Ratio as Indicators of Potential Severity of *Plasmodium vivax* Malaria in Patients with Thrombocytopenia. *PLoS One* (2014) 9(10):e109246.

83. Jain V, Thomas T, Basak S, *et al*. Sequential dysregulated plasma levels of angiopoietins (ANG-2 and ratios of ANG-2/ANG-1) are associated with malaria severity and mortality among hospital admitted cases in South Bastar Region of Chhattisgarh, Central India. *Pathog Glob Health* (2022) 116(1):47–58.

84. Oluboyo AO, Chukwu SI, Oluboyo BO, *et al*. Evaluation of Angiopoietins 1 and 2 in Malaria-Infested Children. *J Environ Public Health* (2020) 2020:2169763.

85. Conroy AL, Phiri H, Hawkes M, *et al*. Endothelium-Based Biomarkers Are Associated with Cerebral Malaria in Malawian Children: A Retrospective Case-Control Study. *PLoS One* (2010) 5(12):e15291.

86. Erdman LK, Dhabangi A, Musoke C, *et al*. Combinations of Host Biomarkers Predict Mortality among Ugandan Children with Severe Malaria: A Retrospective Case-Control Study. *PLoS One* (2011) 6(2):e17440.

87. Adukpo S, Kusi KA, Ofori MF, *et al*. High Plasma Levels of Soluble Intercellular Adhesion Molecule (ICAM)-1 Are Associated with Cerebral Malaria. *PLoS One* (2013) 8(12):e84181.

88. Hyacinth HI, Gee BE, Adamkiewicz T V, *et al*. Plasma BDNF and PDGF-AA levels are associated with high TCD velocity and stroke in children with sickle cell anemia. *Cytokine* (2012) 60(1):302–8.

89. Brouwers J, Noviyanti R, Fijnheer R, *et al*. Platelet Activation Determines Angiopoietin-1 and VEGF Levels in Malaria: Implications for Their Use as Biomarkers. *PLoS One* (2013) 8(6):e64850.

90. Graham SM, Chen J, Chung DW, *et al*. Endothelial activation, haemostasis and thrombosis biomarkers in Ugandan children with severe malaria participating in a clinical trial. *Malar J* (2016) 15(1):1–9.

91. Phiri HT, Bridges DJ, Glover SJ, *et al*. Elevated Plasma Von Willebrand Factor and Propeptide Levels in Malawian Children with Malaria. *PLoS One* (2011) 6(11):e25626.

92. Barber BE, William T, Grigg MJ, *et al*. Parasite Biomass-Related Inflammation, Endothelial Activation, Microvascular Dysfunction and Disease Severity in *Vivax* Malaria. *PLoS Pathog* (2015) 11(1):e1004558.
